# Supplementary figures and images for: The ratio of adaptive to innate immune cells differs between genders and associates with improved prognosis and response to immunotherapy
Source: PLoS One. 2023 Feb 6;18(2):e0281375. doi: 10.1371/journal.pone.0281375 (PMC9901741; doi:10.1371/journal.pone.0281375)

a

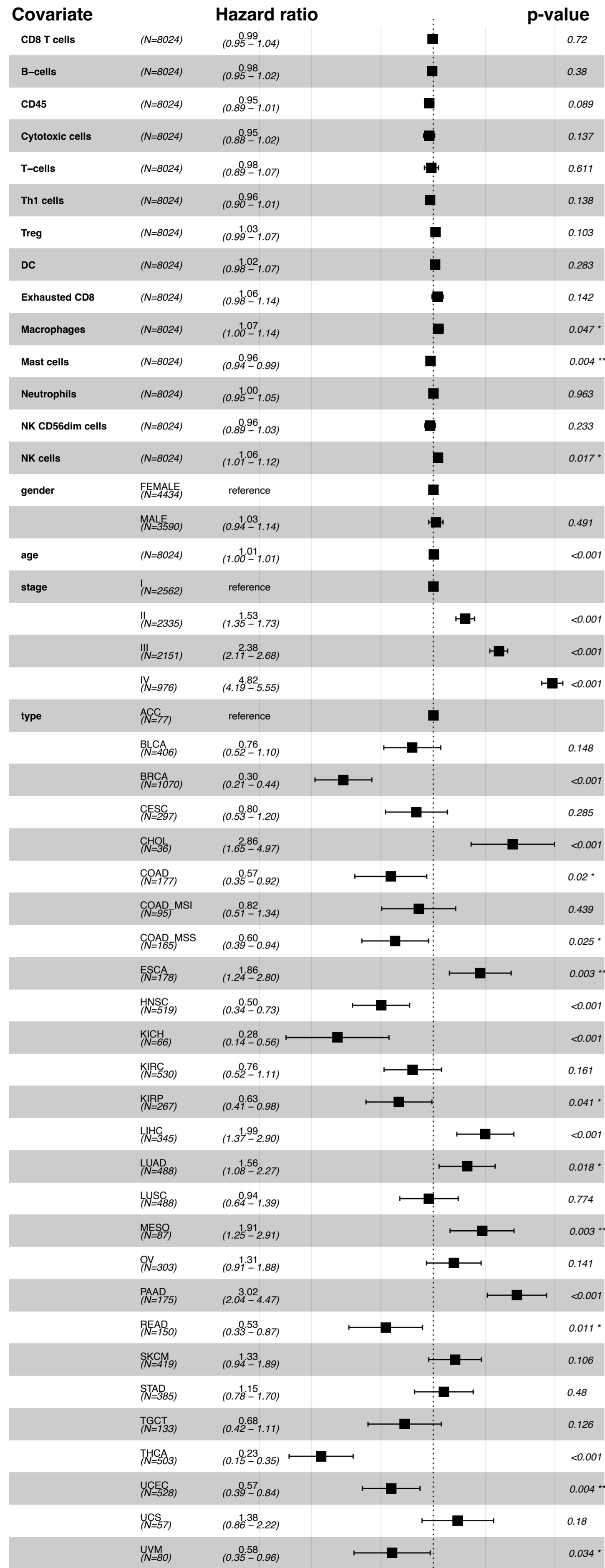

# Events: 2655; Global p-value (Log-Rank): 0  
AIC: 42438.16; Concordance Index: 0.74

b

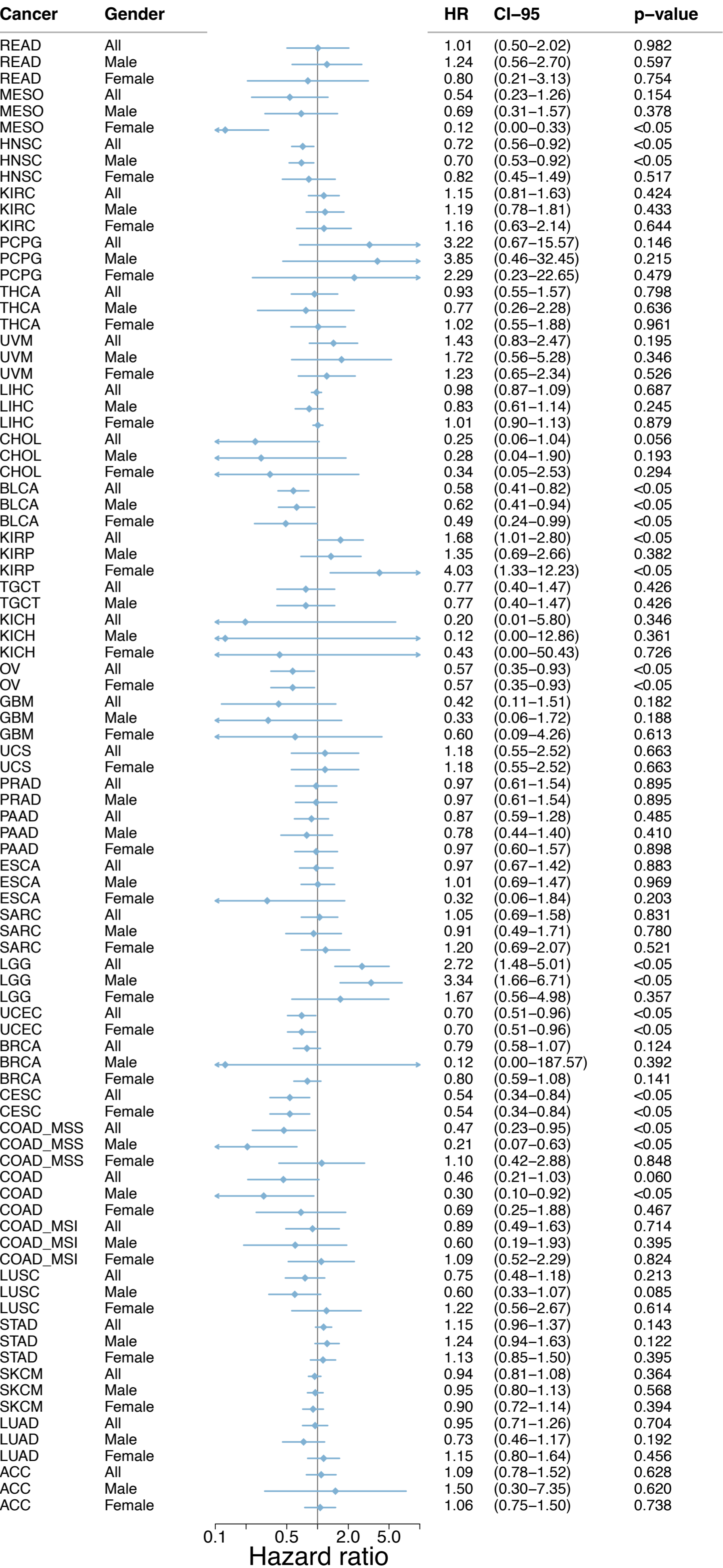

Supplement: S1 Fig — a) A forest plot showing the hazard ratio from a multivariate cox proportional hazard regression for progression of cancer for the expression of each of the cell types in the TIL calculation, gender, age, stage and cancer types as covariates. b) A forest plot showing the hazard ratio from a univariate cox proportional hazard regression for progression of cancer. A univariate model was done for each cancer type and for both genders within the cancer type individually. (PDF) [file pone.0281375.s001.pdf]

AI ratio

Female  
Male

8  
4  
2  
1  
0.5  
0.25  
0.125

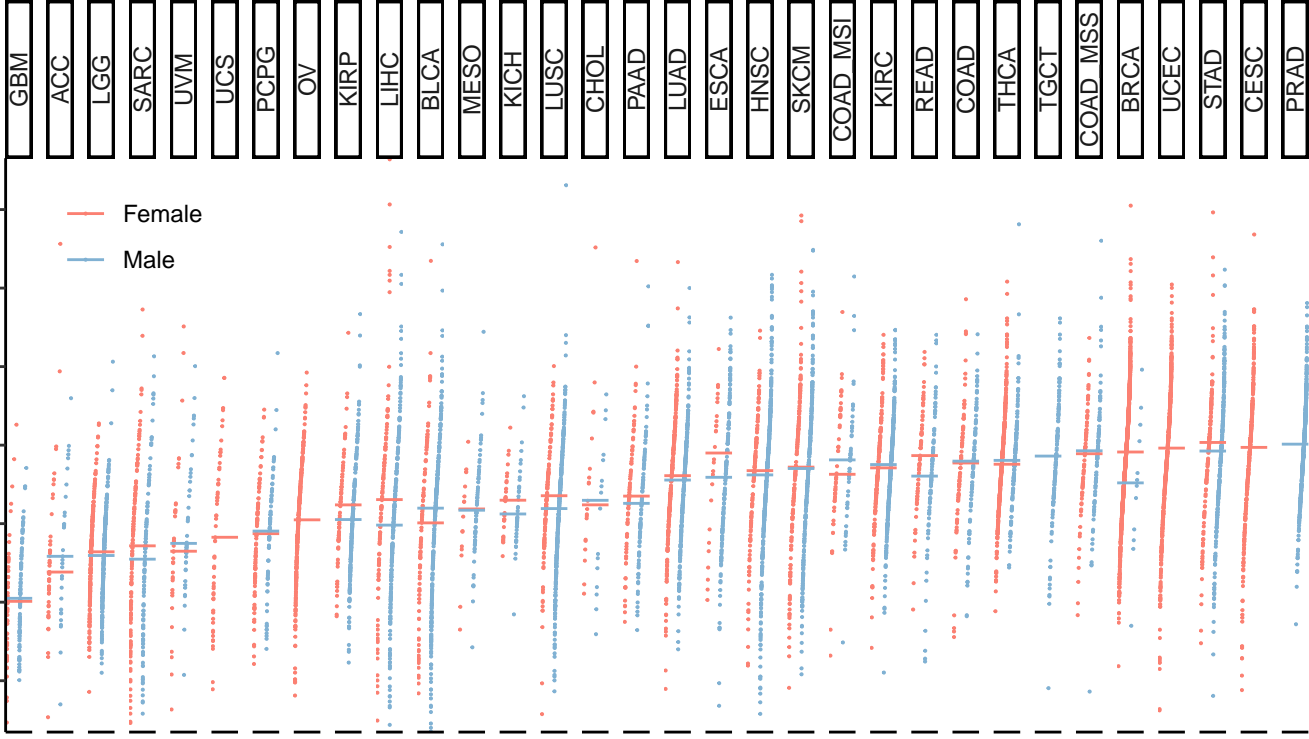

Supplement: S2 Fig — The A/I ratio for 29 cancer types in the TCGA cohort. The cancertypes are ordered by median A/I ratio, female and male patients are represented by red and blue dots respectively, the median for each gender in each cancer type is represented by a horizontal line. (PDF) [file pone.0281375.s002.pdf]

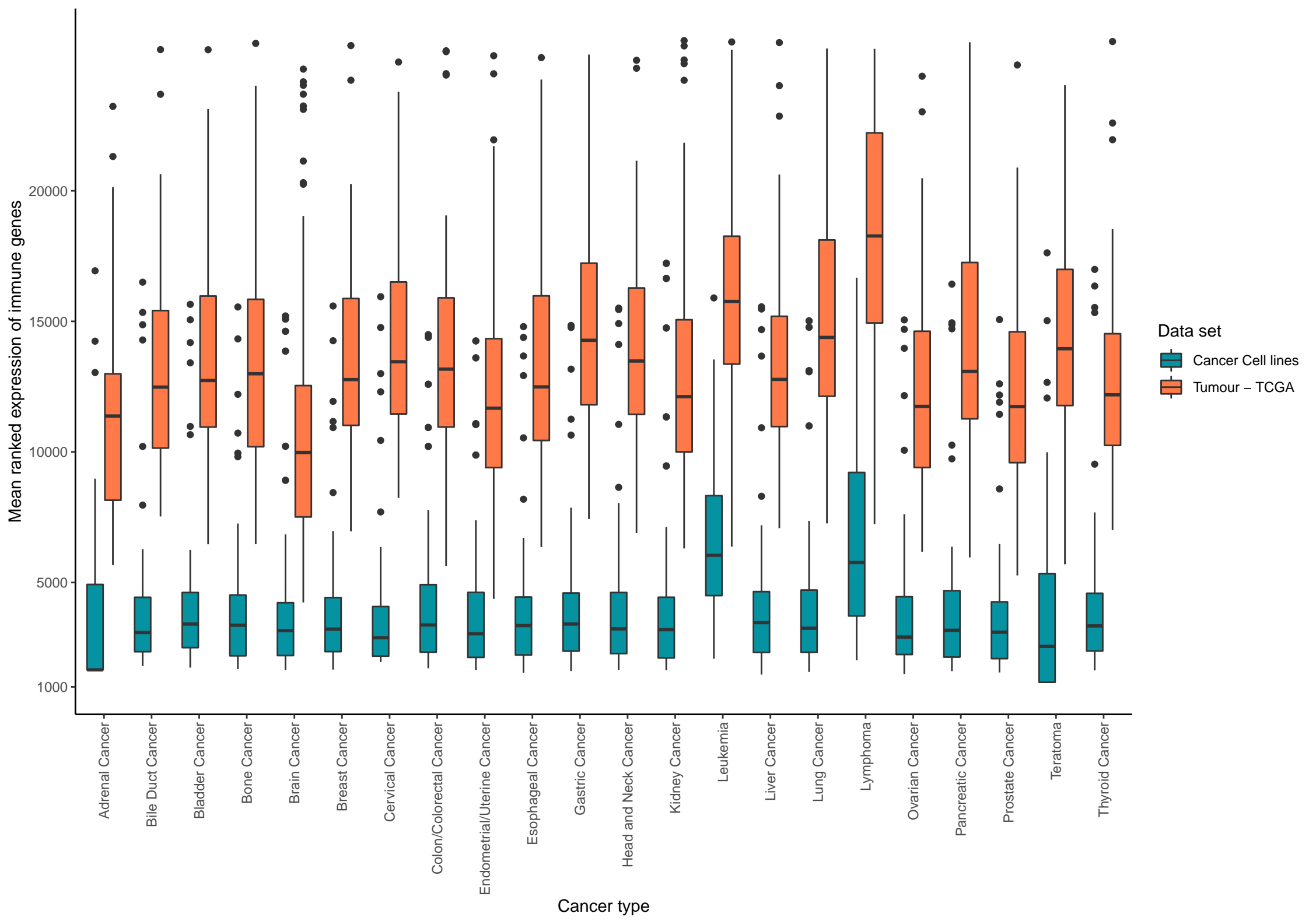

Supplement: S3 Fig — The mean ranked expression per cancer type of the 67 immune related genes that the celltype scores are calculated from, for both the cancer cell line data from the CCLE project, and from the tumour samples in TCGA. Low rank = low expression. (PDF) [file pone.0281375.s003.pdf]

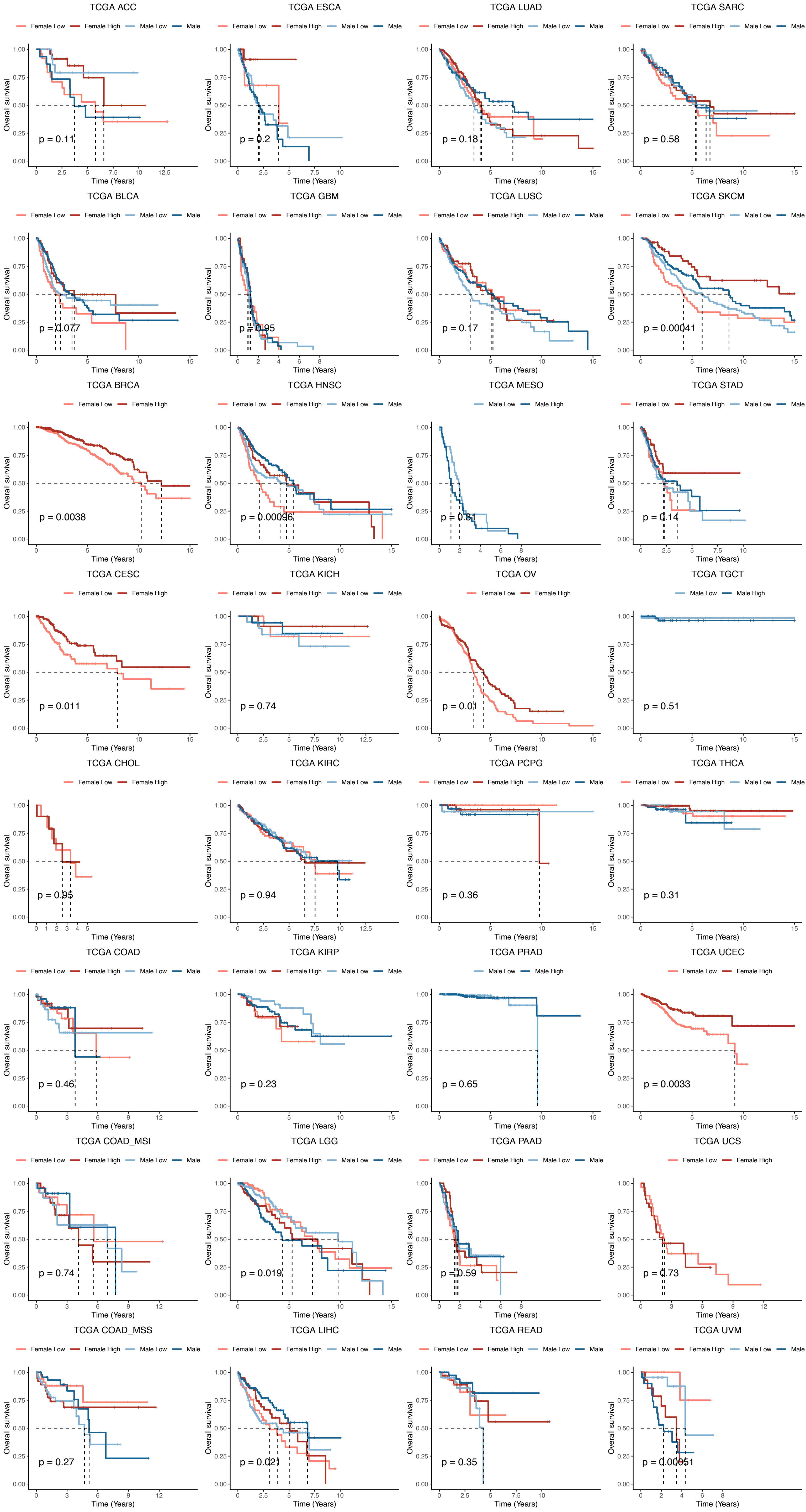

Supplement: S4 Fig — Kaplan-Meier curves showing the 15-year survival for each cancer type within the TCGA cohort, the patients are stratified by gender and A/I ratio. (PDF) [file pone.0281375.s004.pdf]

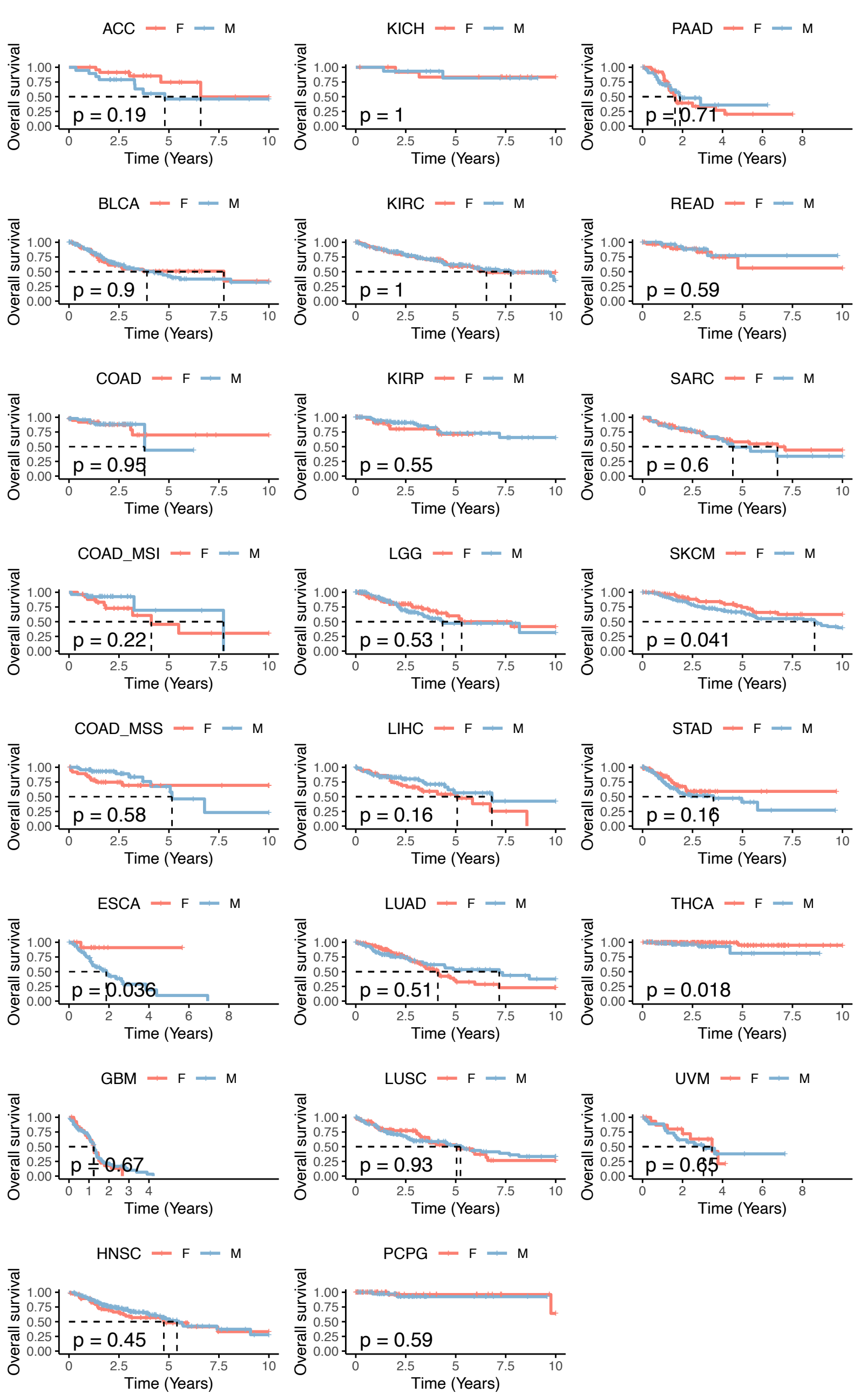

Supplement: S5 Fig — Kaplan-Meier curves showing the 10-year survival for TCGA patients with an A/I score above the female median, Male vs. Females. A p-value for the difference in survival is available for each cancer type. (PDF) [file pone.0281375.s005.pdf]

HMF BLCA

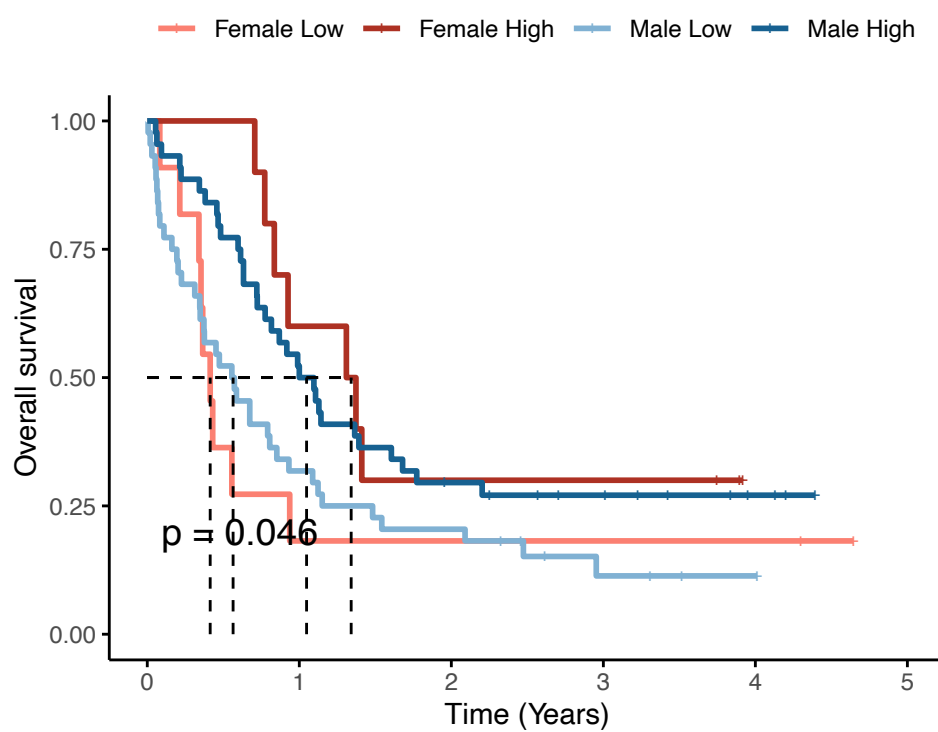

HMF KIRC

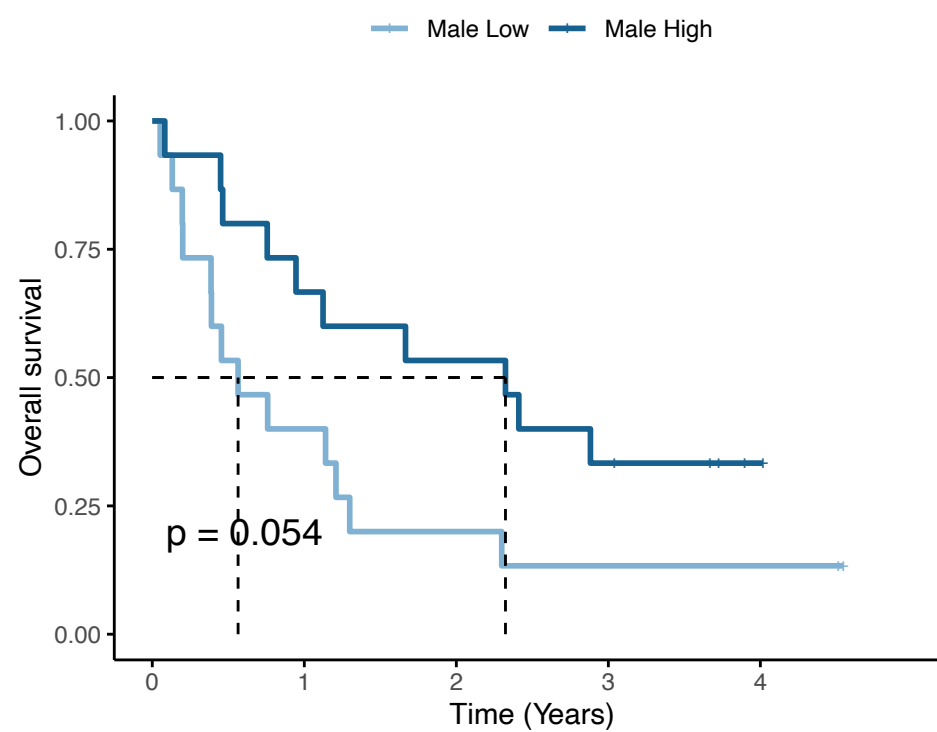

HMF SARC

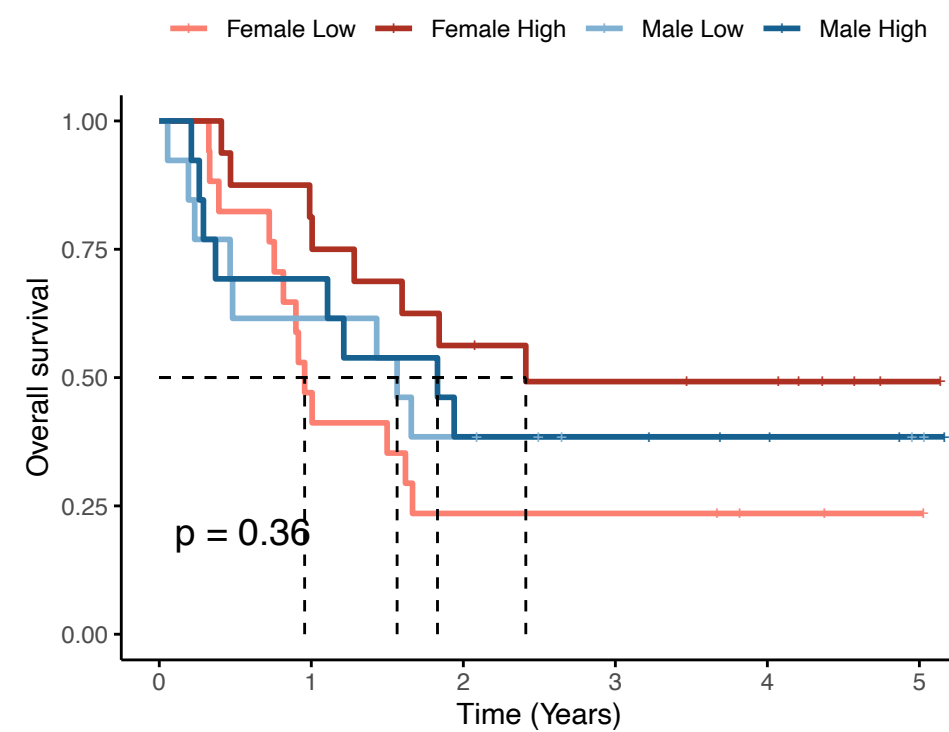

HMF BRCA

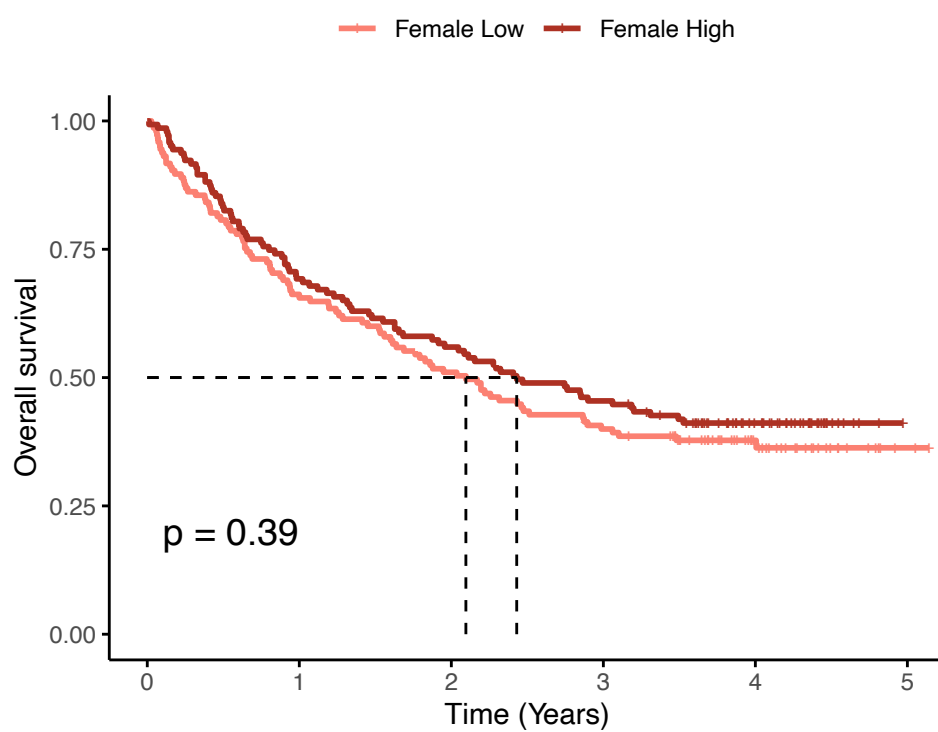

HMF LUNG

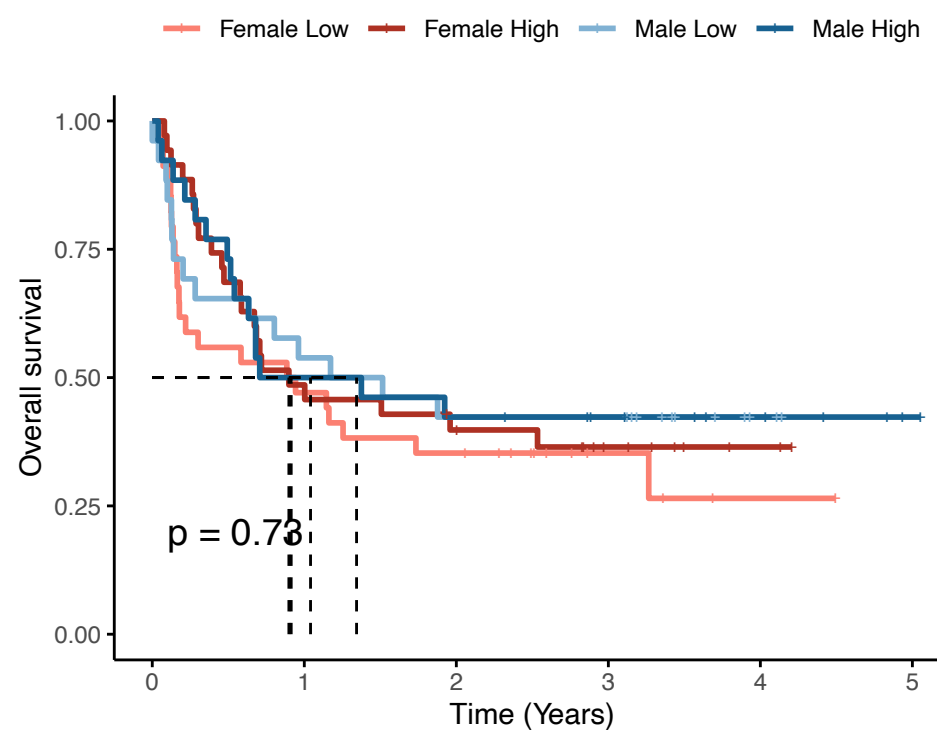

HMF SKCM

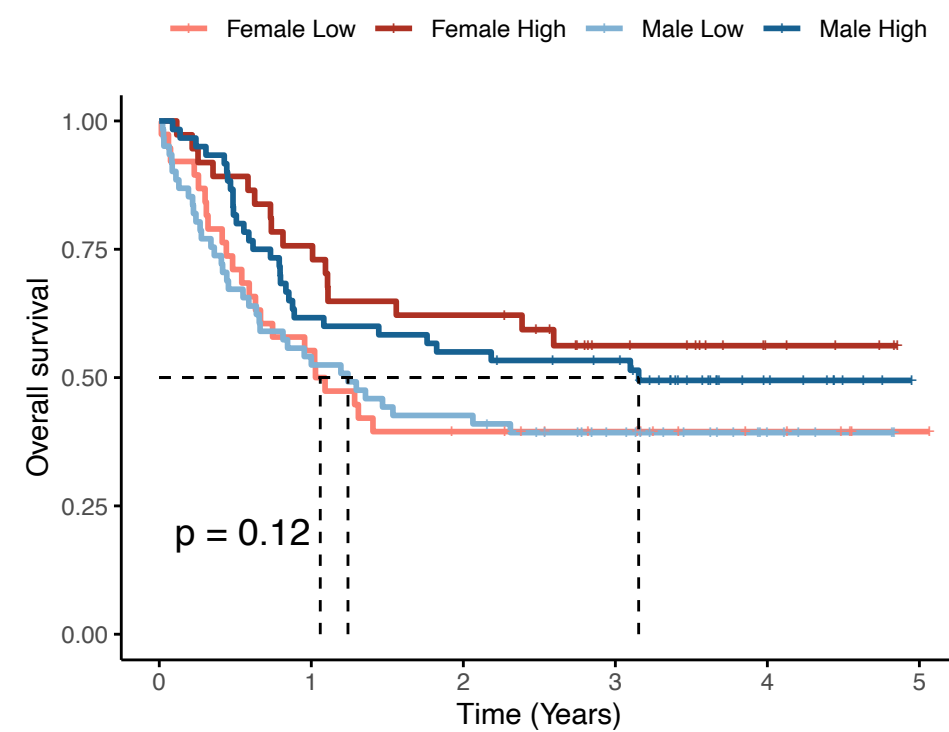

HMF COAD

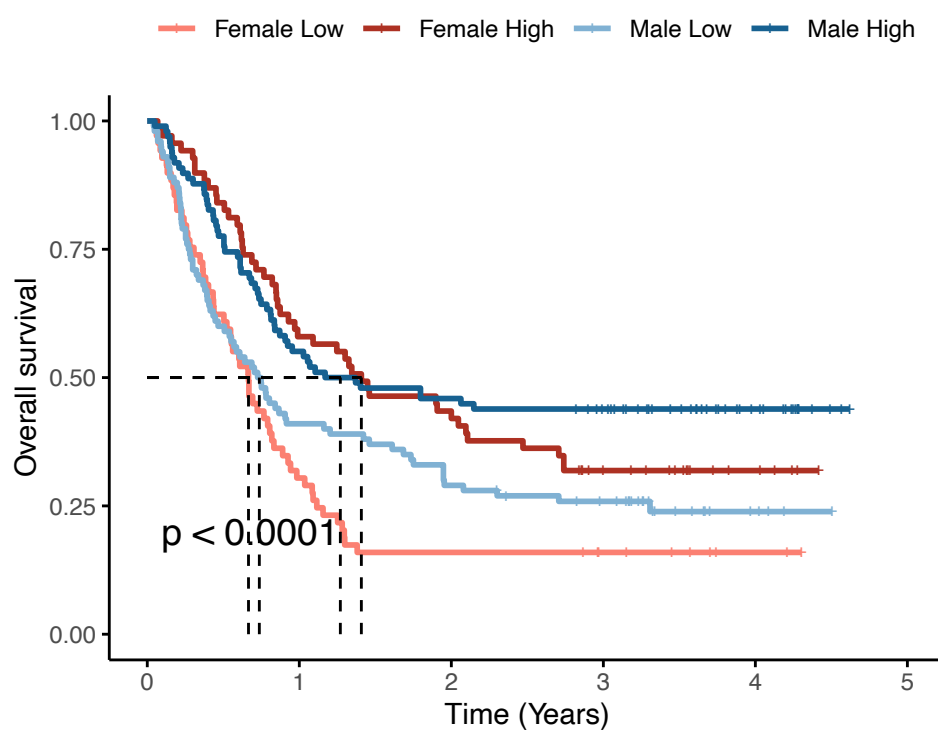

HMF OV

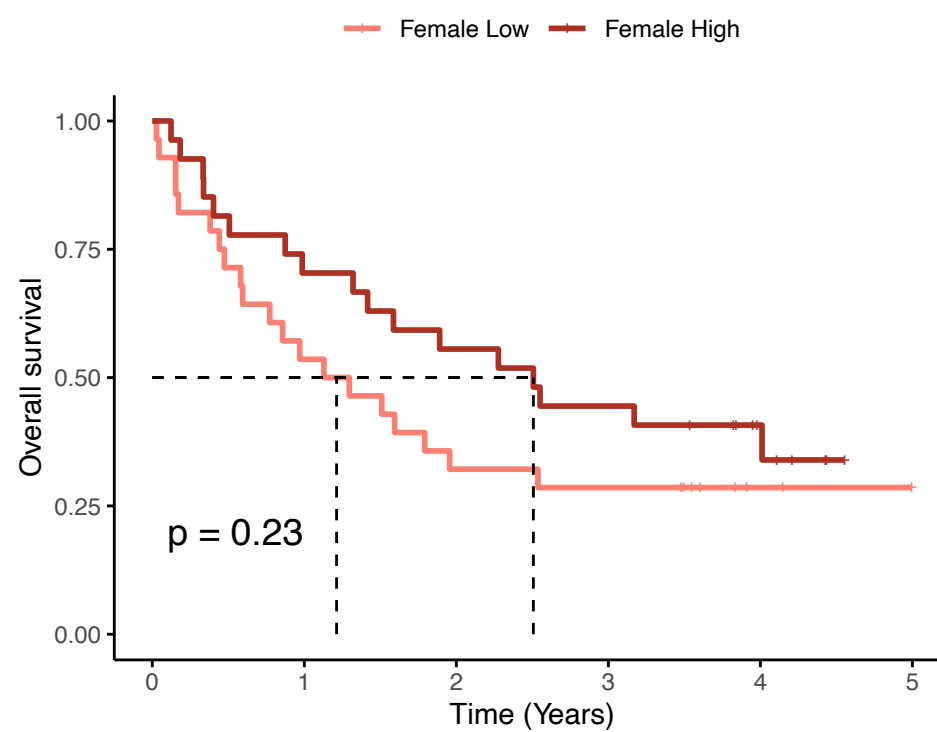

HMF PRAD

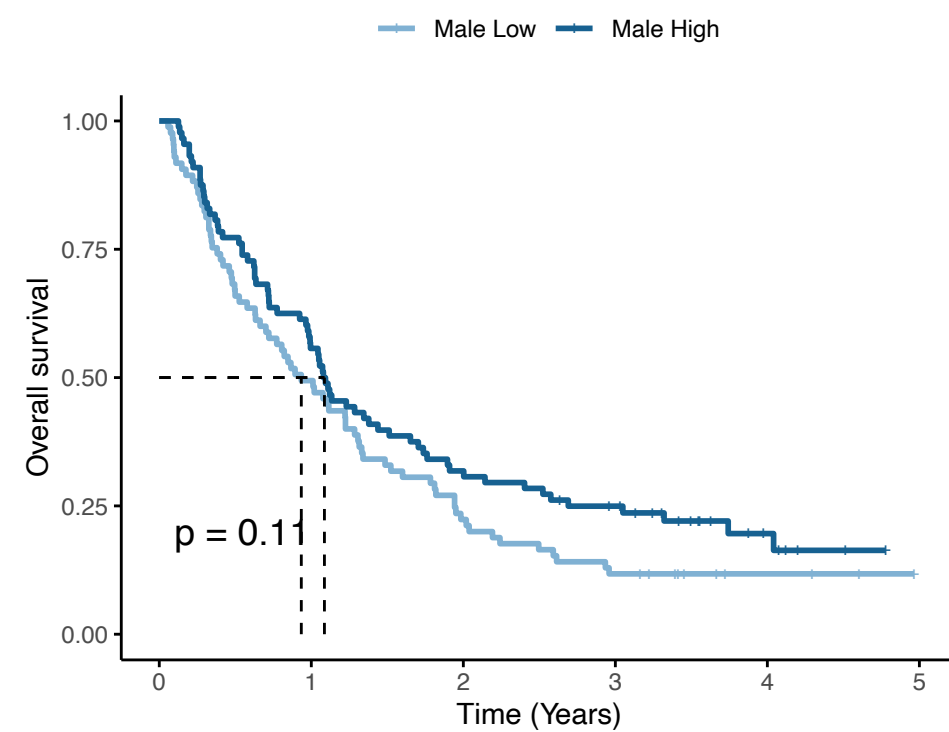

HMF ESCA

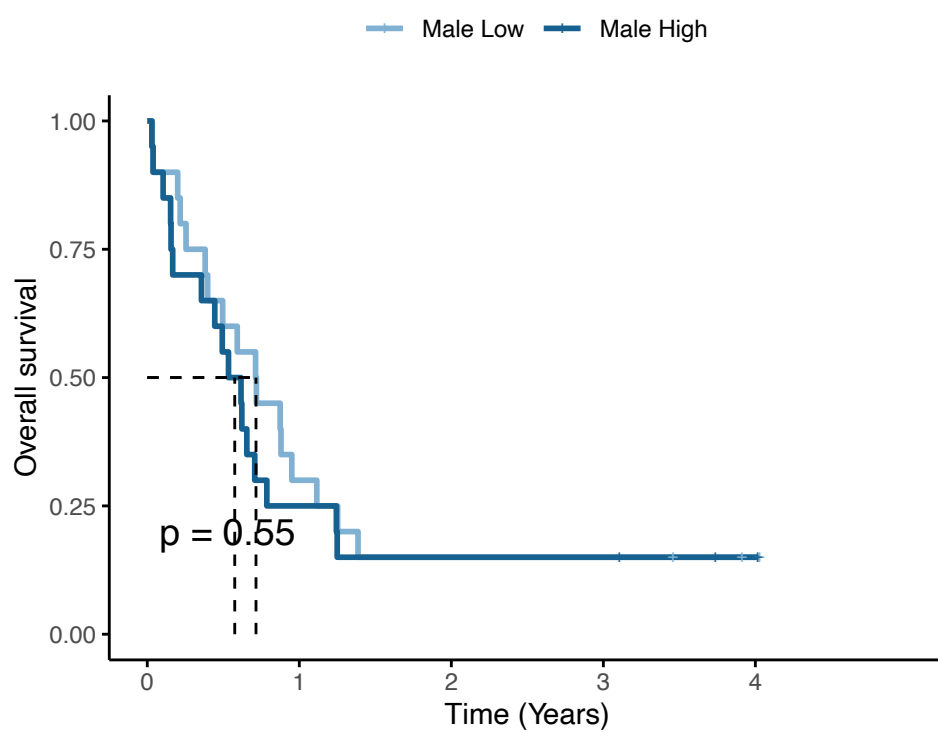

HMF PAAD

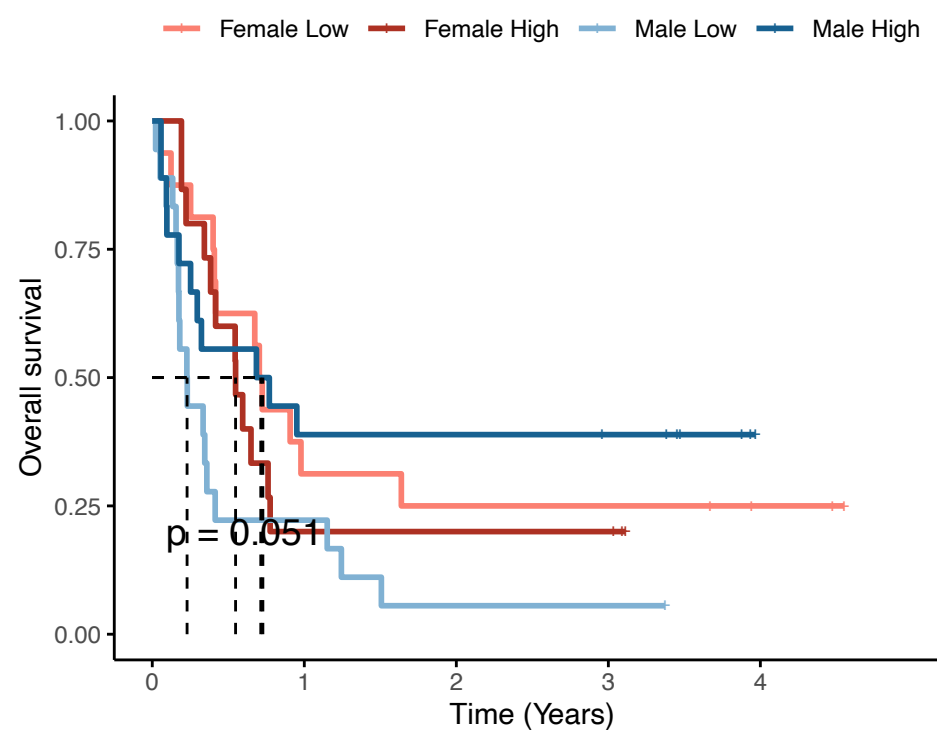

Supplement: S6 Fig — Kaplan-Meier curves showing the five-year survival for each cancer type within the HMF cohort, the patients are stratified by gender and A/I ratio. (PDF) [file pone.0281375.s006.pdf]

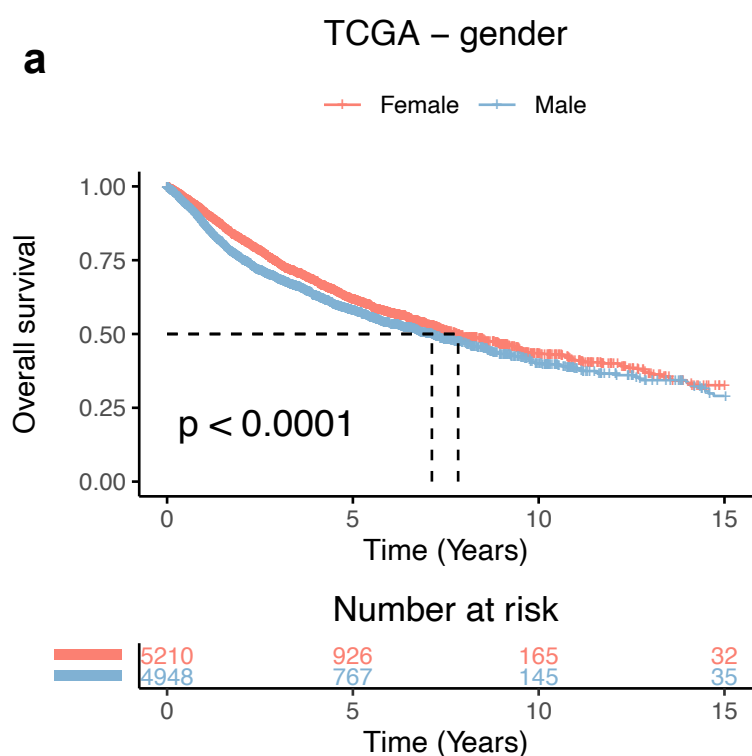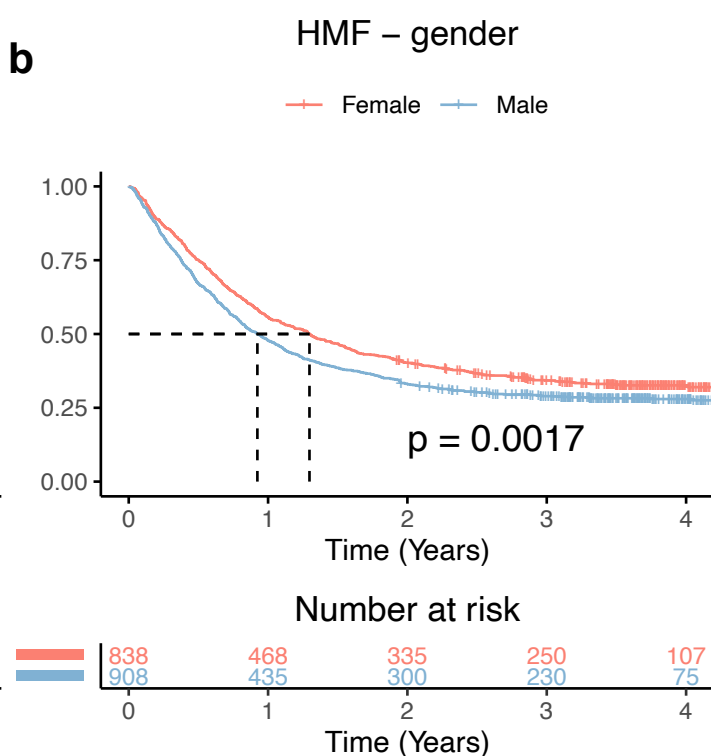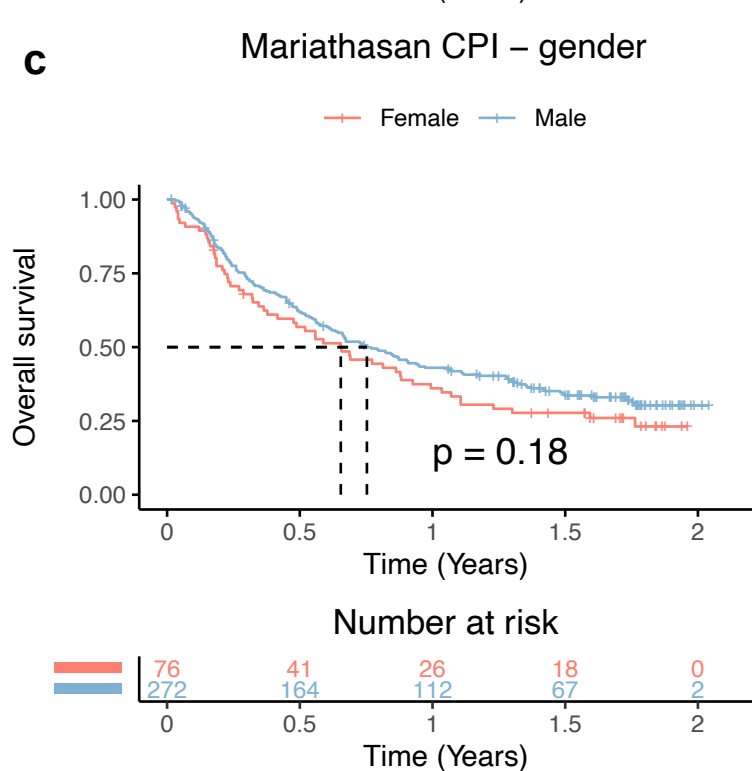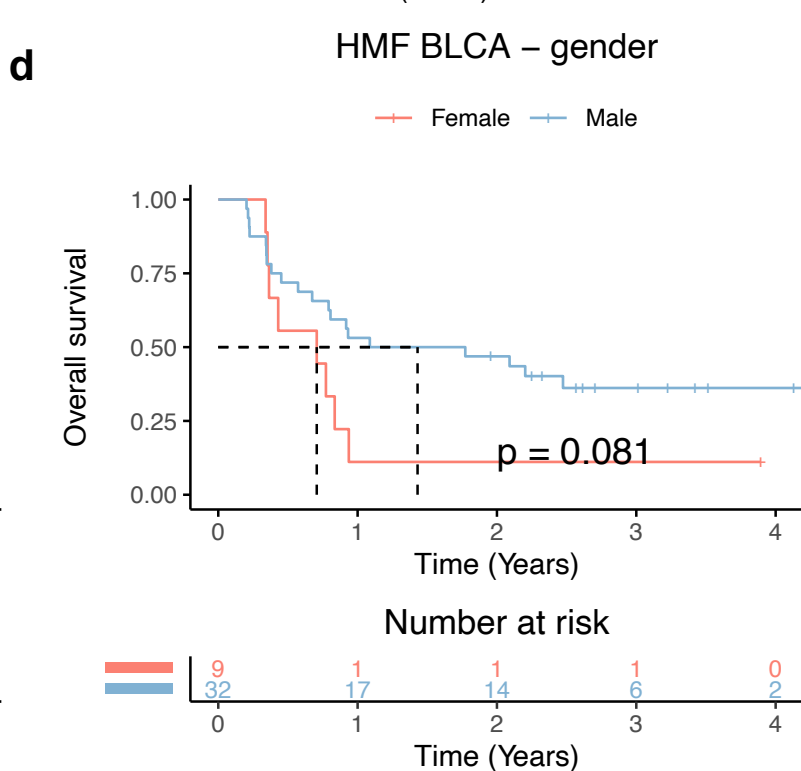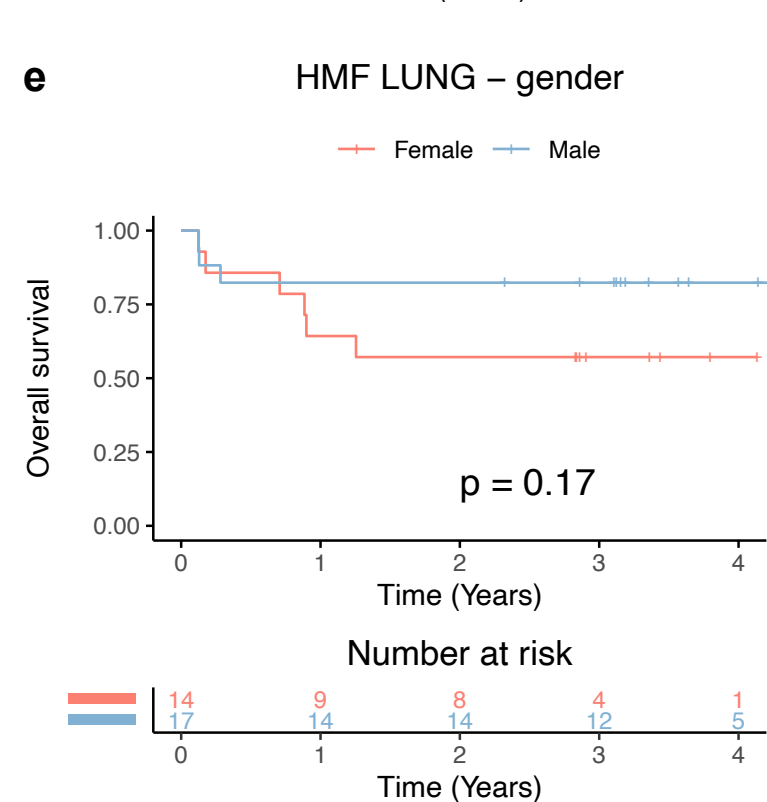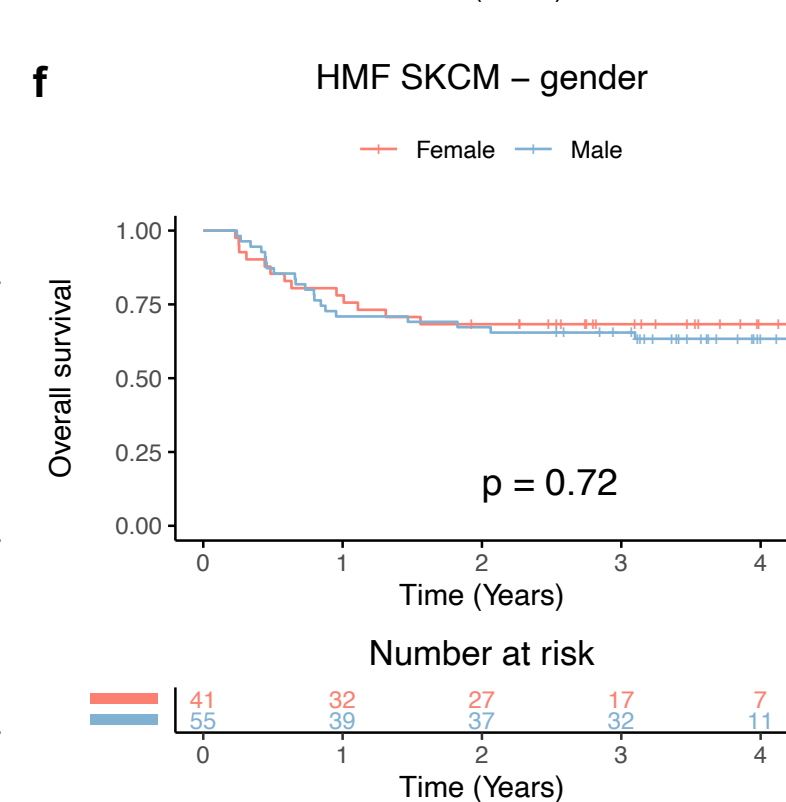

Supplement: S7 Fig — a) Kaplan-Meier curve of 10,158 patients from the TCGA dataset. b) Kaplan-Meier curve of 1746 patients from the HMF dataset. c) Kaplan-Meier curve of 348 patients from the Mariathasan bladder cancer dataset, all CPI treated. d) Kaplan-Meier curve of CPI treated patients from the HMF BLCA dataset. e) Kaplan-Meier curve of CPI treated patients from the HMF LUNG dataset. f) Kaplan-Meier curve of CPI treated patients from the HMF SKCM dataset. (PDF) [file pone.0281375.s007.pdf]

**a**

Mariathasan

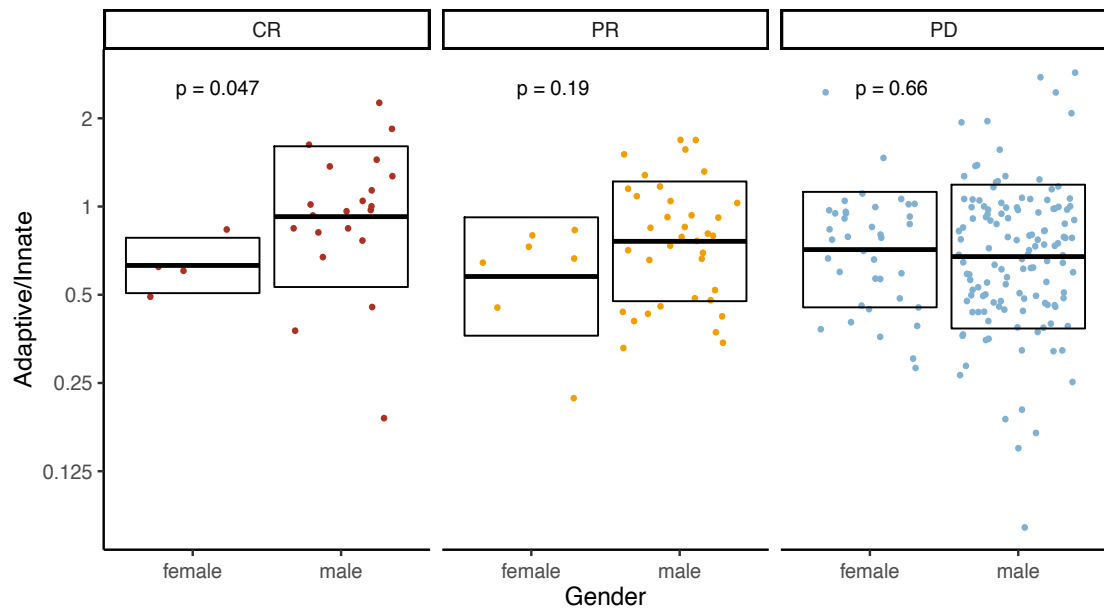**b**

HMF

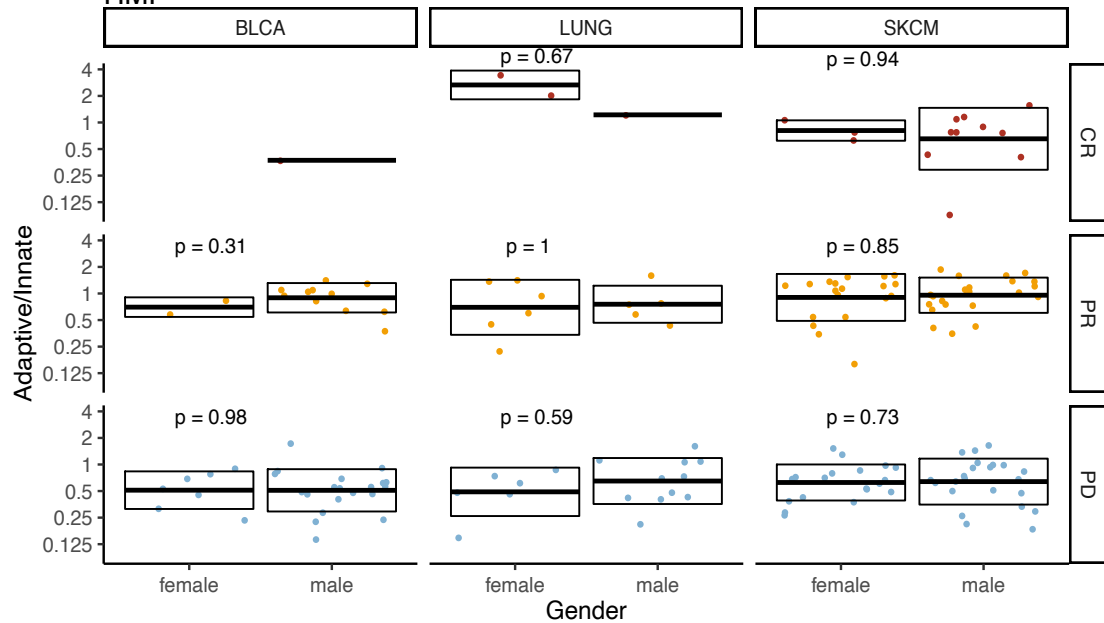

Supplement: S8 Fig — a) Gender stratified response to immunotherapy for the Mariathasan dataset. P-value for difference between gender for each category. b) Gender stratified response to immunotherapy for the HMF dataset, separated by cancer type. P-value for difference between gender for each category. (PDF) [file pone.0281375.s008.pdf]

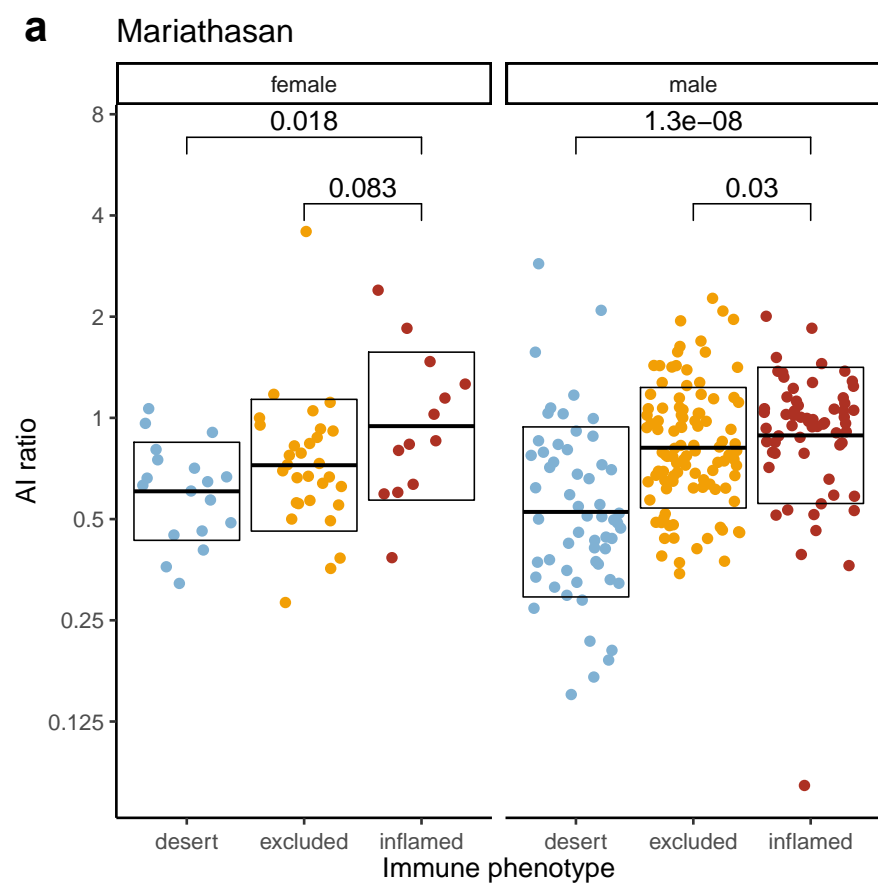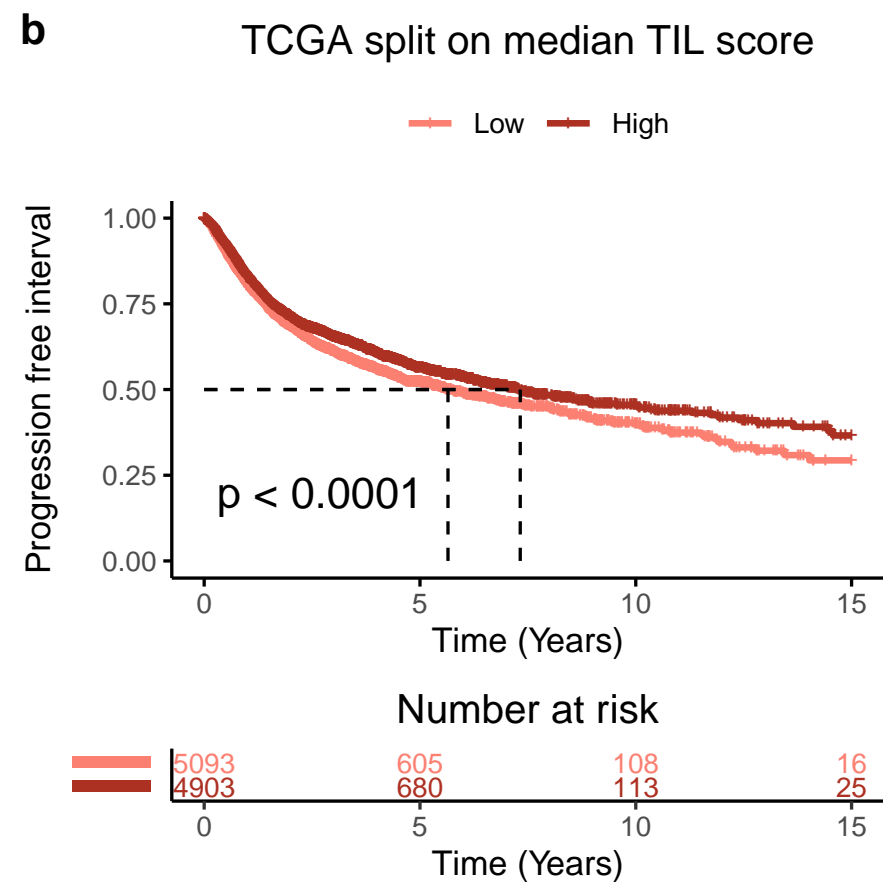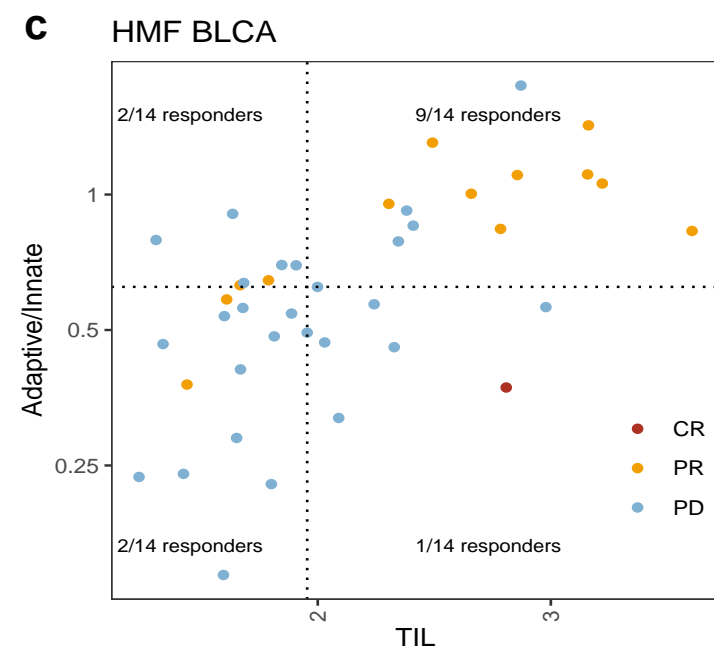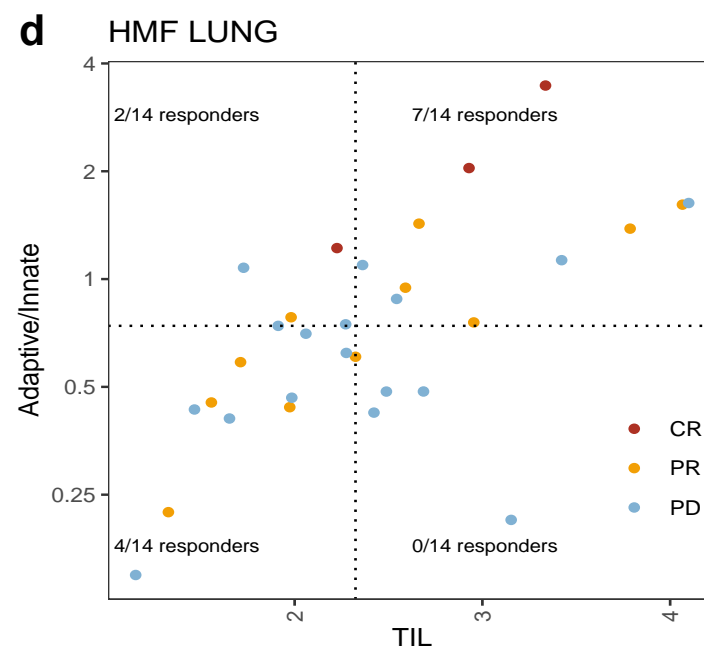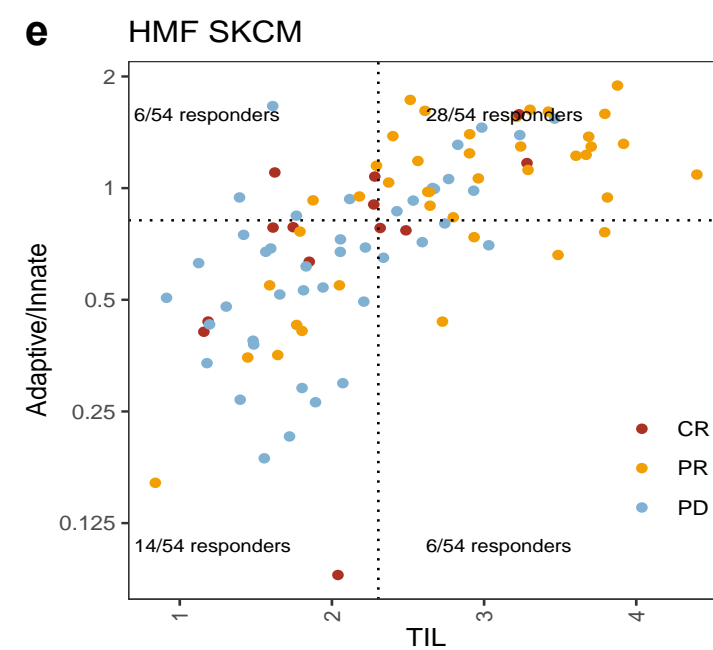

Supplement: S9 Fig — a) The A/I ratio vs. the immune phenotype for the Mariathasan dataset, separated by gender. P-values are for comparisons between inflamed vs. desert and inflamed vs. excluded. b) Kaplan-Meier curve for survival of TCGA patients with a high or low TIL score. c-e) A/I ratio vs TIL score for the CPI treated patients separated by cancer type, coloured by response category. In the respective cancer types (BLCA: P = 0.003, LUNG: P = 0.15, SKCM: P = 0.006). (PDF) [file pone.0281375.s009.pdf]

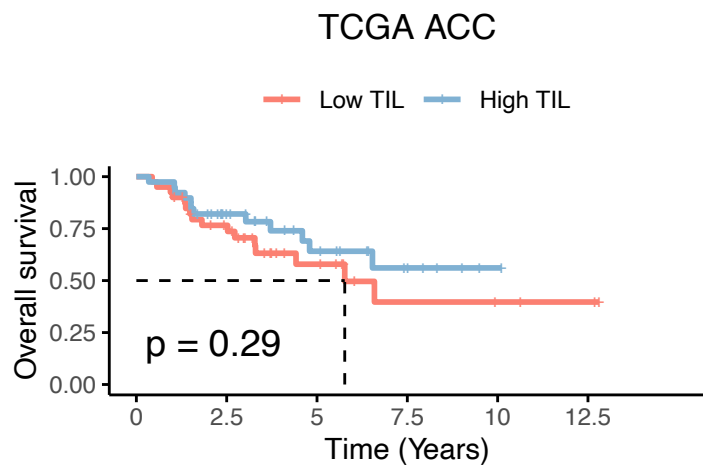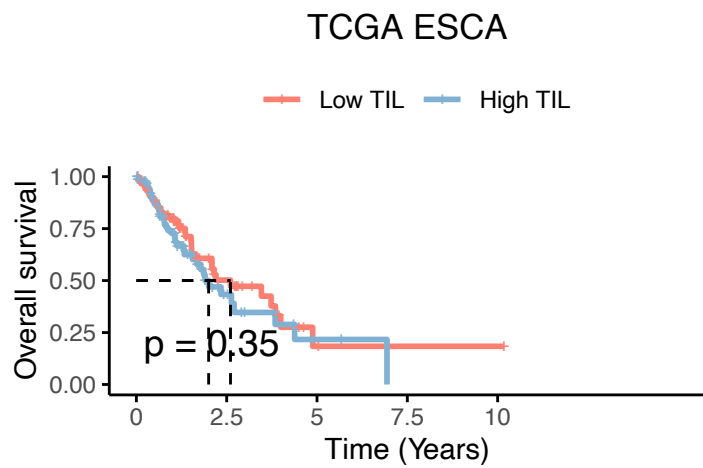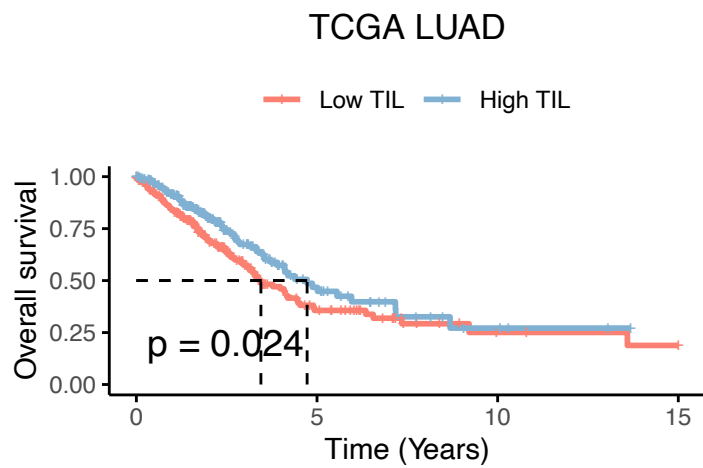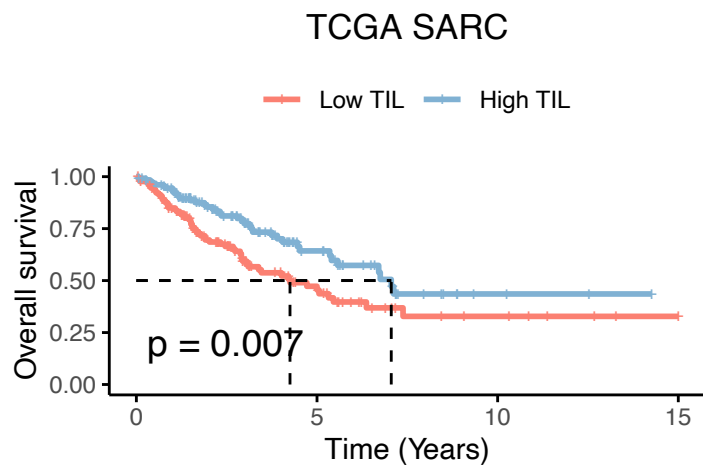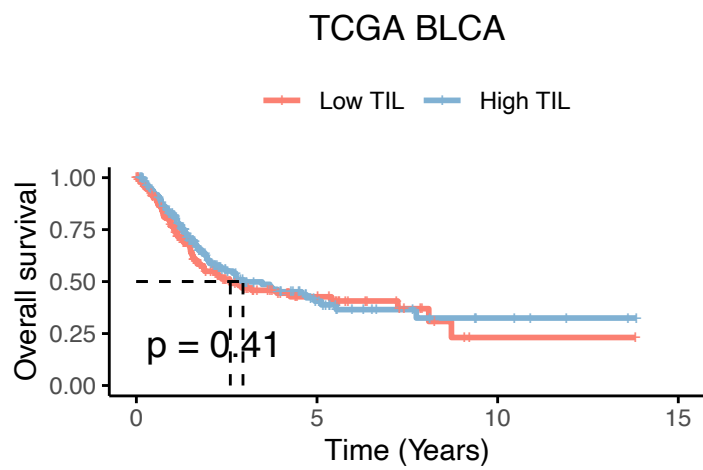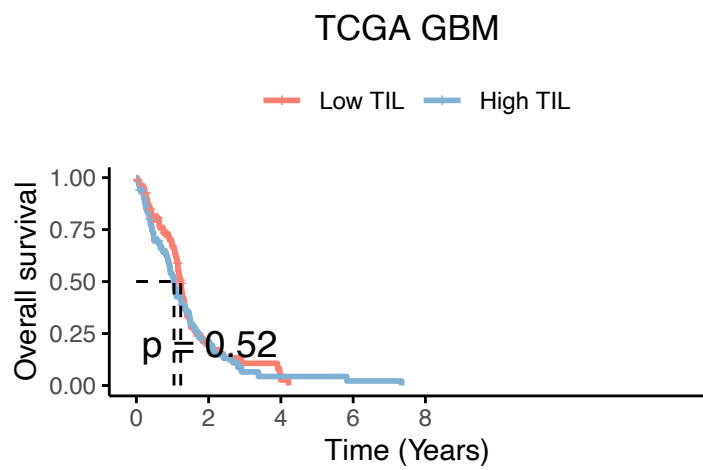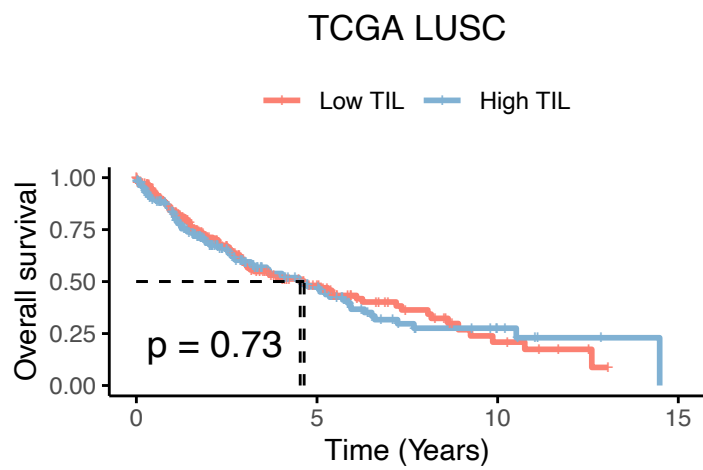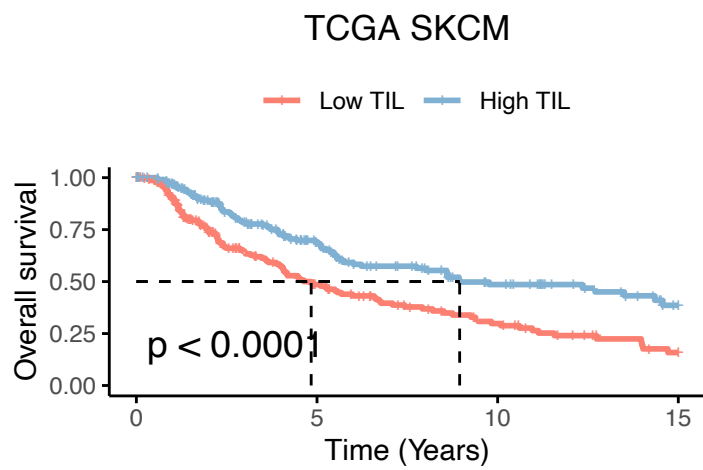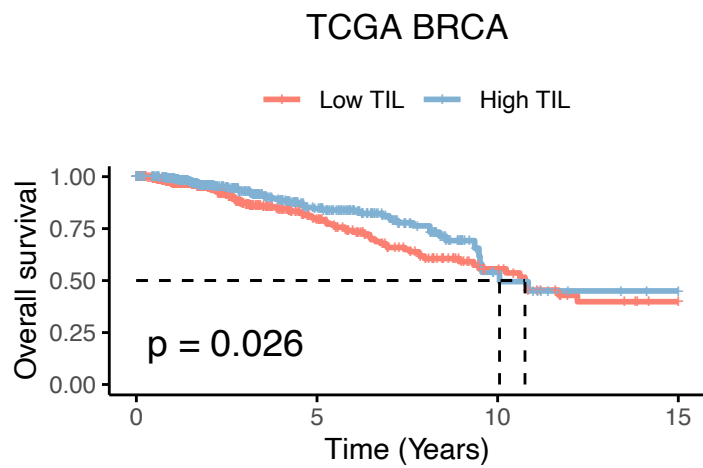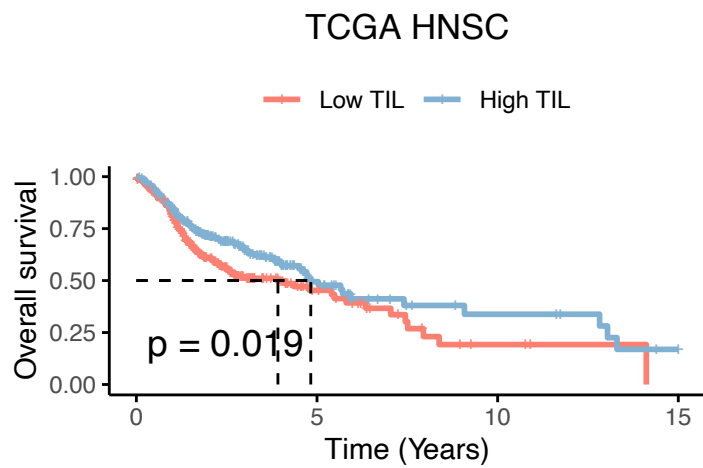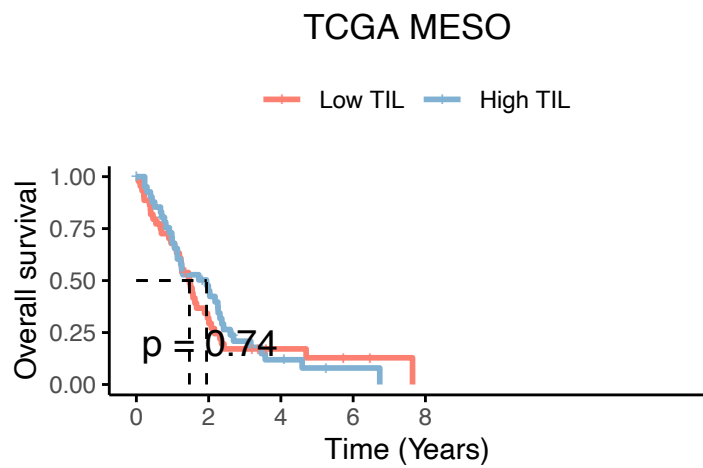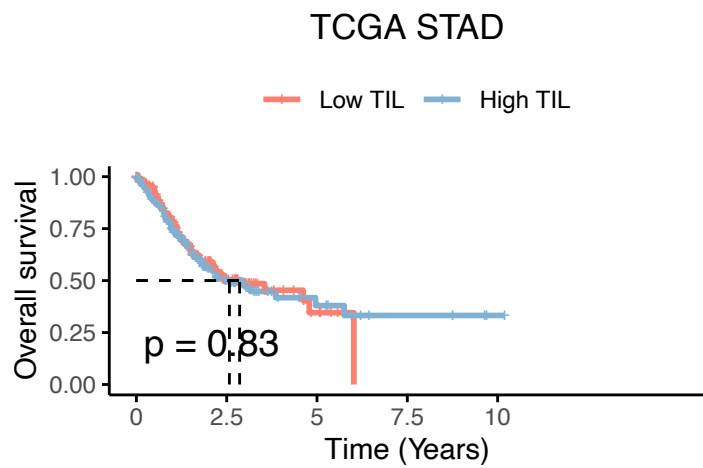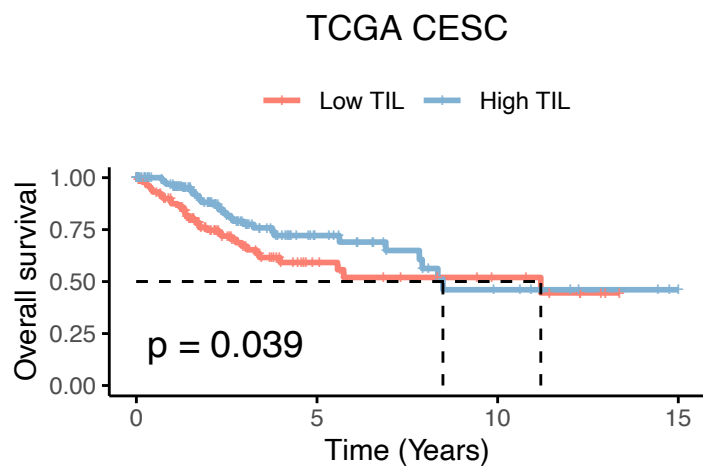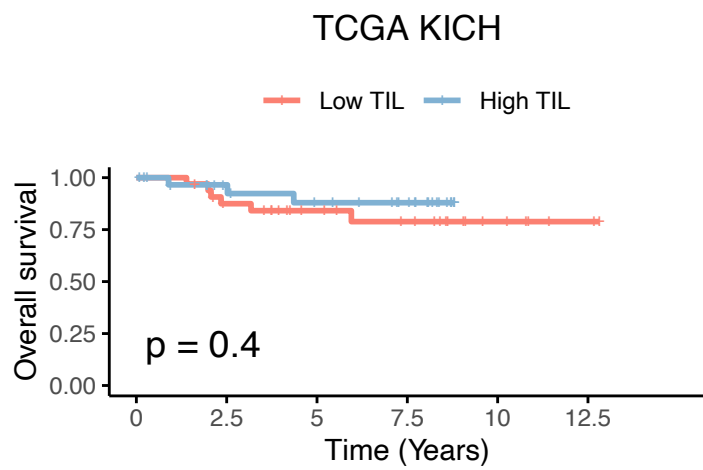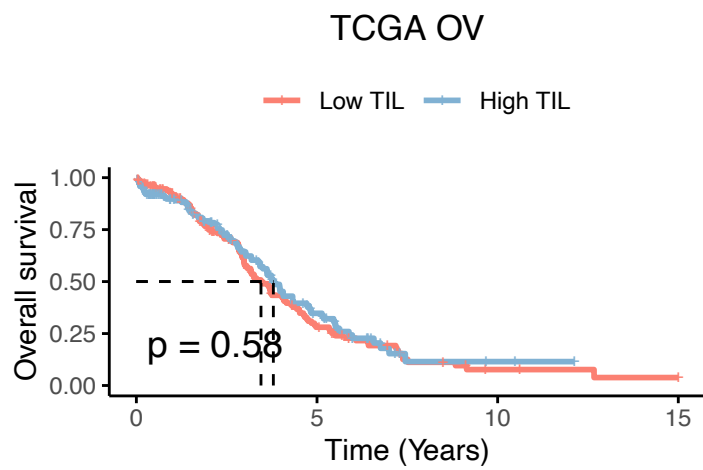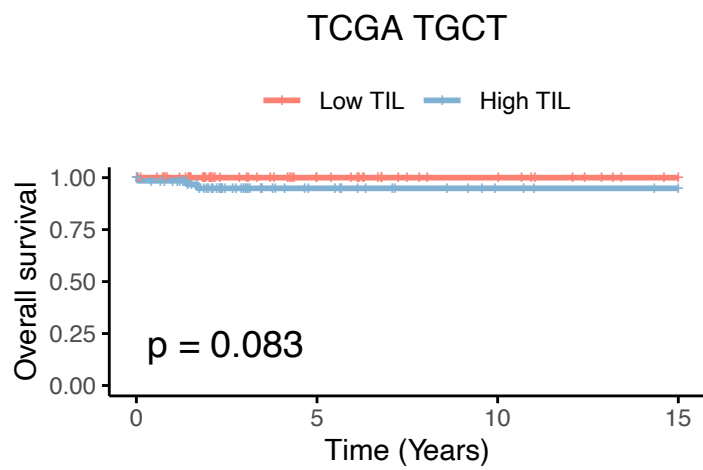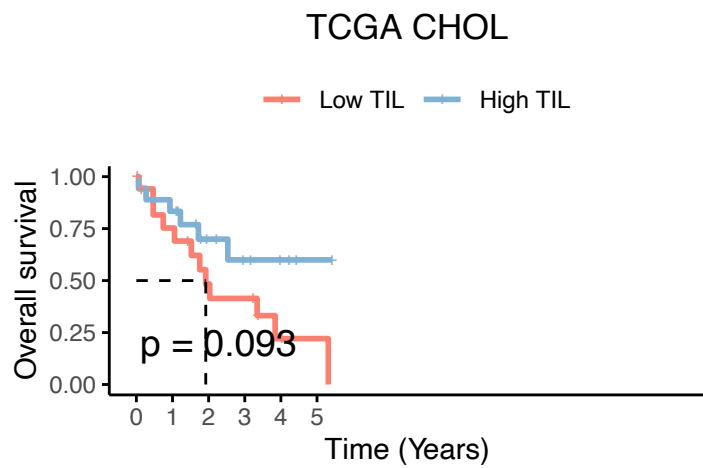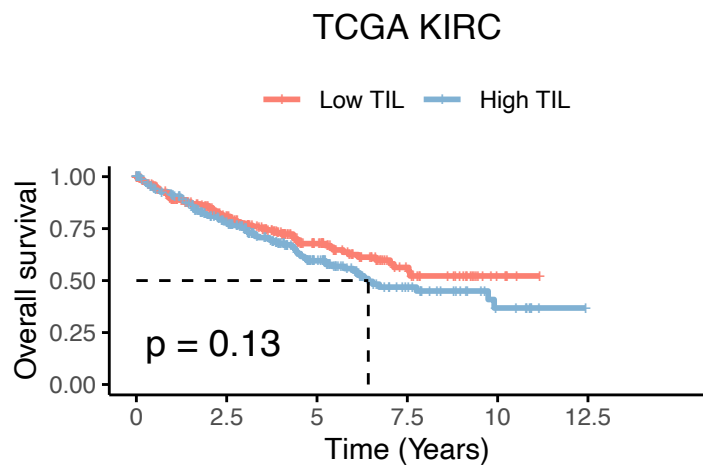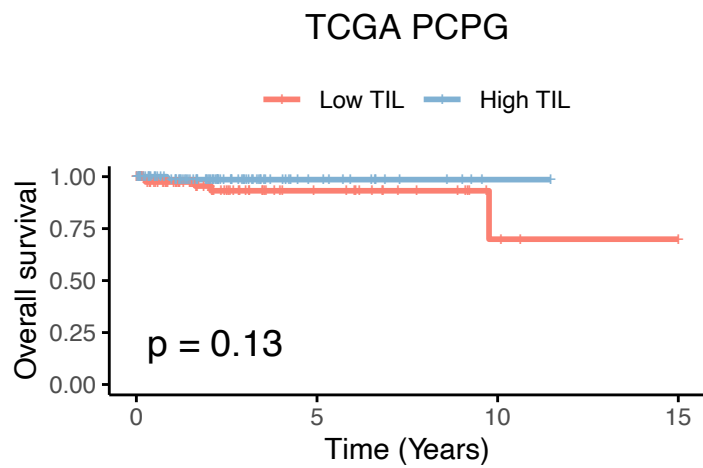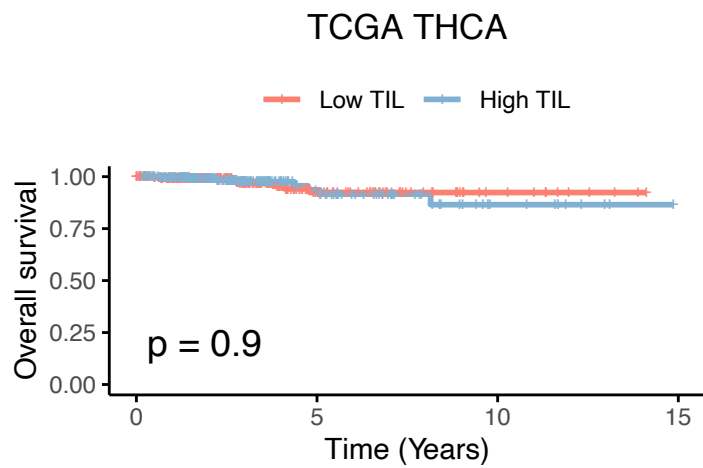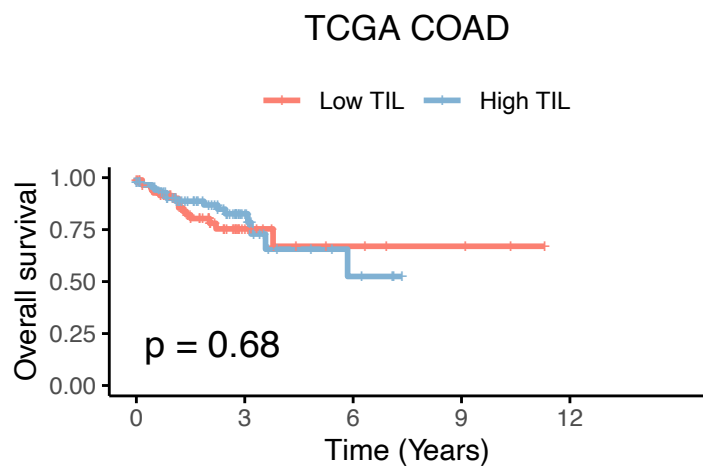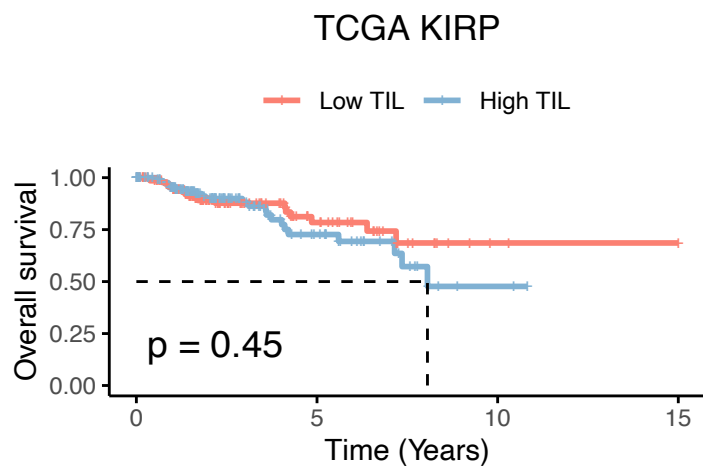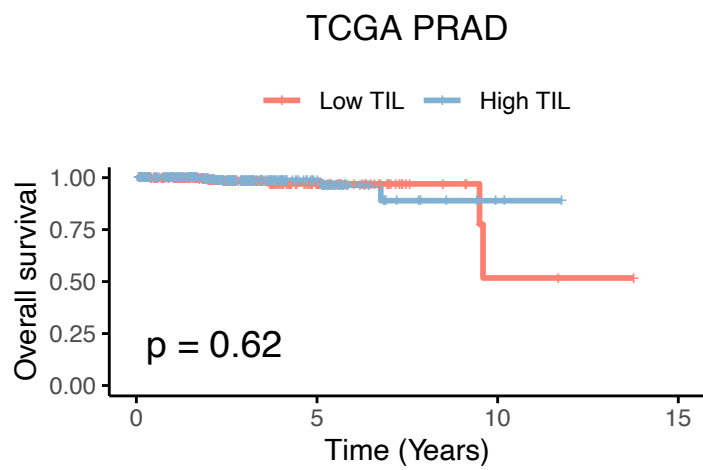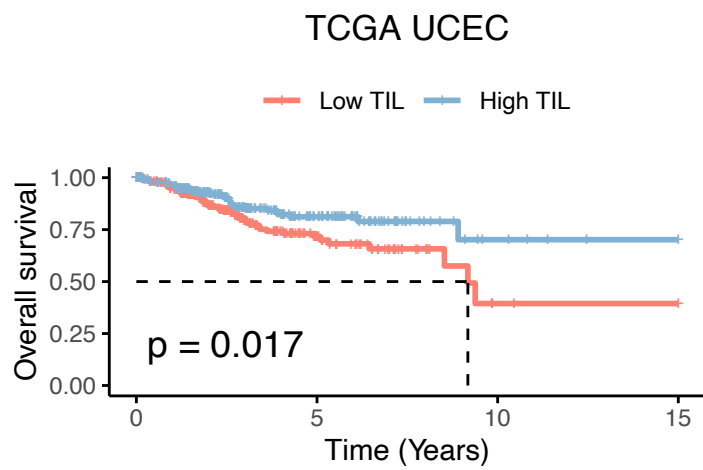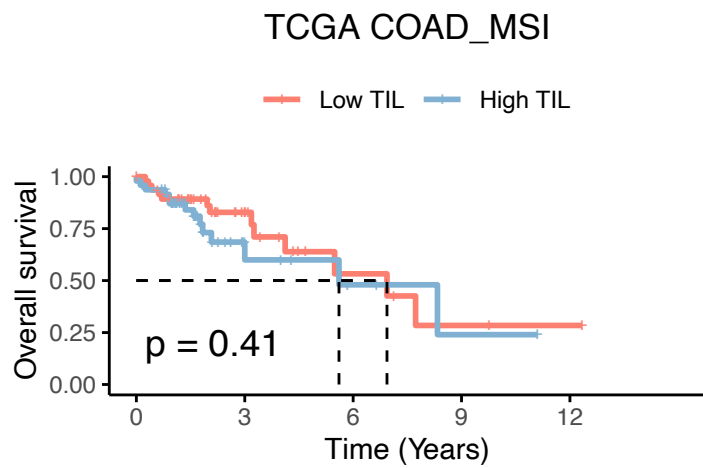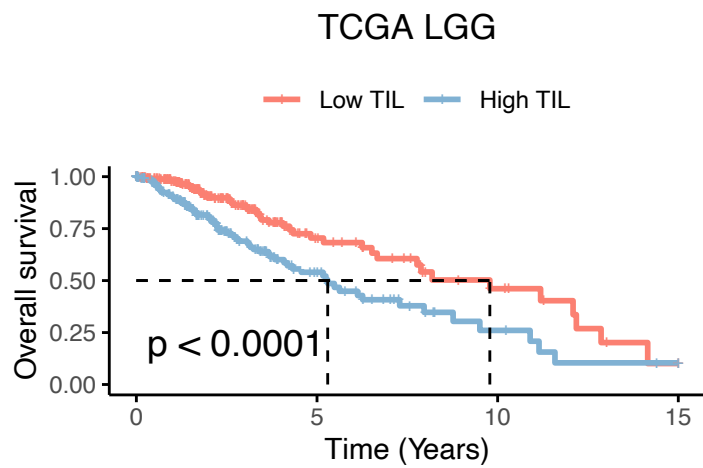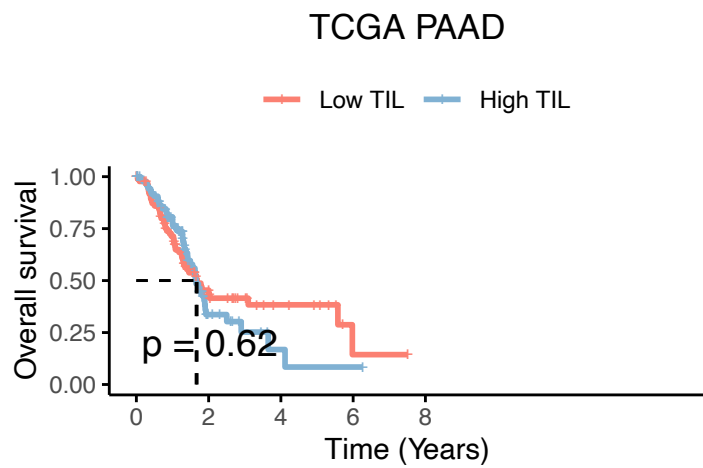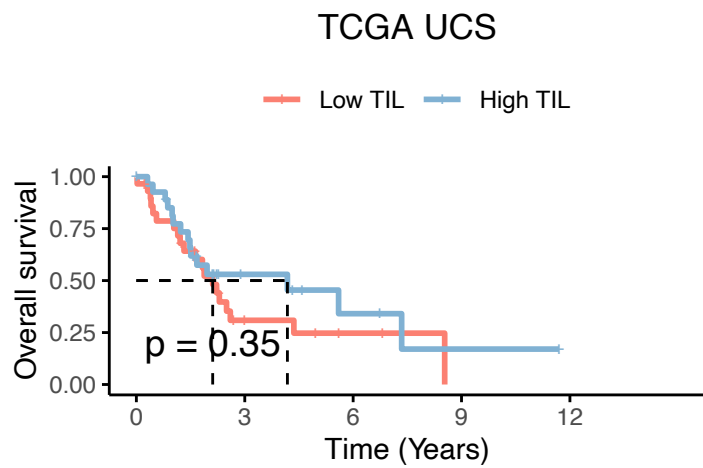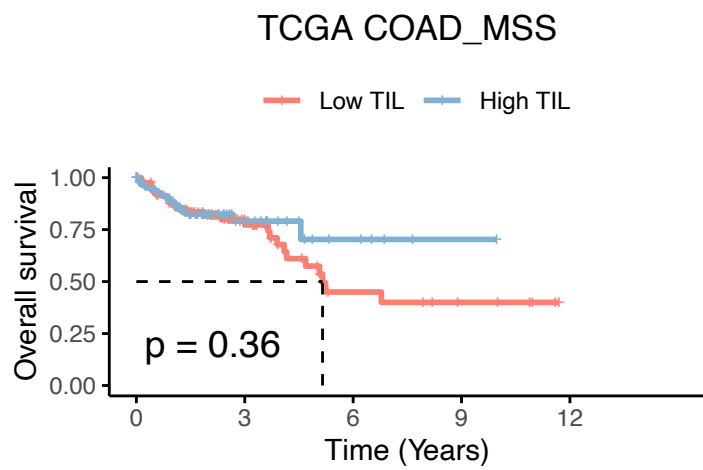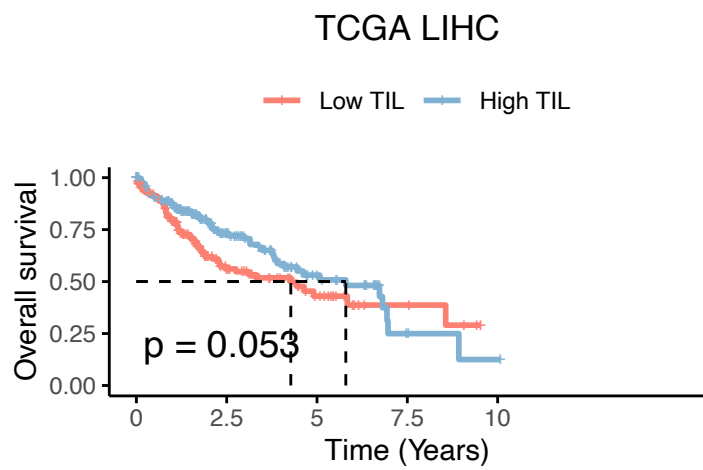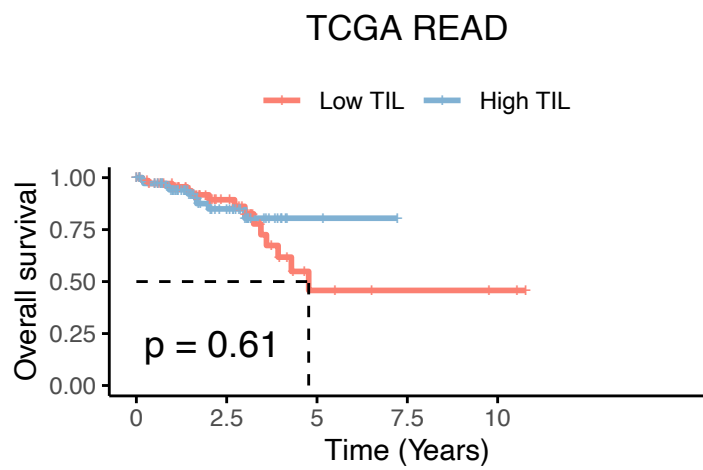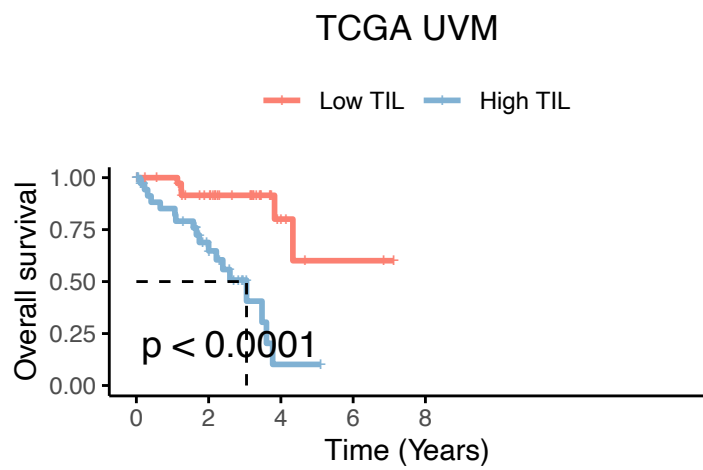

Supplement: S10 Fig — Kaplan-Meier curves showing the 15-year survival for each cancer type within the TCGA cohort, the patients are stratified by gender and TIL score. (PDF) [file pone.0281375.s010.pdf]

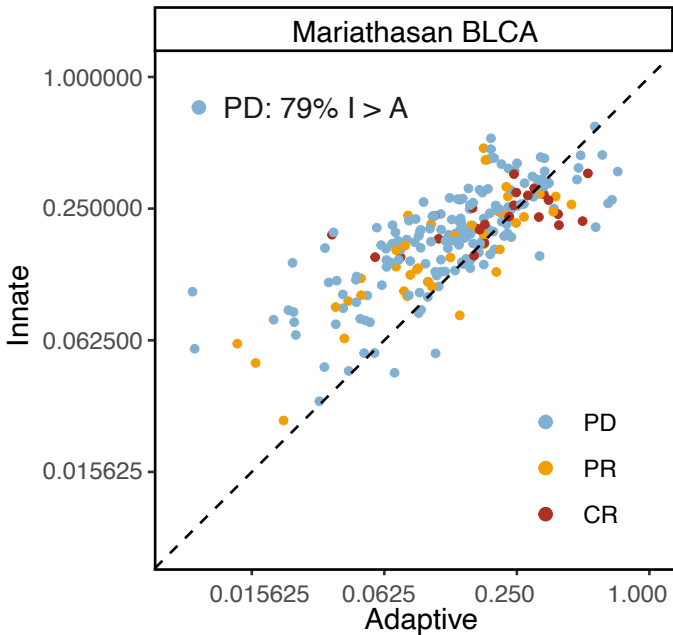

Supplement: S11 Fig — Scatterplots showing the adaptive immune expression vs. the innate immune expression in each patient for the Mariathasan cohort. The points are coloured by their response to immunotherapy. (PDF) [file pone.0281375.s011.pdf]
