# Supplementary material for: The ratio of adaptive to innate immune cells differs between genders and associates with improved prognosis and response to immunotherapy
Source: PLoS One. 2023 Feb 6;18(2):e0281375. doi: 10.1371/journal.pone.0281375 (PMC9901741; doi:10.1371/journal.pone.0281375)
Supplement: Attachment — Submitted filename: Rebuttal_letter.pdf [file pone.0281375.s015.pdf]

Reviewer #1: Thank you for addressing my comments; I agree the manuscript is stronger now. It's got many interesting results, presented well.
Before publication, I have to insist: the manuscript should not include any results from pan-cancer analyses ignoring cancer type. Because of the very strong confounding by tumor type, the results of these analyses are misleading. The detailed comments call out results of this nature. To be clear: I consider all these detailed comments to be essential and will not support publication unless they are addressed.

We appreciate the reviewers excellent comments, particularly we note the reviewer agrees with us that the manuscript has been significantly improved following the initial review and with incorporation of the reviewers excellent suggestions into the text and figure layout.

However, we politely disagree with the firm notion that pan-cancer analysis is not a valid scientific approach. Cancer is inherently a genomic disease inflicted upon host cells, all sharing the *exact* same genome initially. The site of origin is critical for the clinical trajectory of the disease, but different cancer types are not fundamentally different diseases, as may be the case for diseases caused by external pathogens, viruses and bacteria. Indeed, pan-cancer analysis may reveal common characteristics in the body's natural defenses against cancer, but also differences likely dictated by the tissue microenvironments and cellular programming depending on the tissue of origin.

Indeed, pan-cancer analysis is not novel. In the scientific literature, pan-cancer analysis is commonly found already. Eg., in this manuscript by Combes and colleagues, published in Cell earlier this year, the authors deployed a similar type of analysis, as shown in figure 3b:

https://www.sciencedirect.com/science/article/pii/S0092867421014264

And for further examples:
Figure 3a:
https://cancerci.biomedcentral.com/articles/10.1186/s12935-021-02266-3

Figur 8;
https://www.nature.com/articles/s41389-019-0121-7

Figur 1:
https://www.mdpi.com/2072-6694/11/10/1562/htm

We accept that the reviewer may disagree on this approach, however in our humble opinion that does not invalidate the analysis but can be presented as a weakness. To alleviate this concern, we have now included both pan-cancer and cancer-type specific analysis throughout the manuscript, and a specific section in the discussion:

"While we have found a highly significant association between the A/I ratio and patient outcome , both in pan-cancer analysis and in a meta-analysis across cancer types, the A/I ratio was not found to be significantly associated for all cancer types individually. A part of this may be explained by limited power in some cancer types, where there are not enough patients within each group to reach statistical significance, despite a supporting trend. However, undoubtedly cancer-type specific differences also play a role. It is all but certain that the A/I ratio, despite being a systemic marker rather than a cancer-specific marker, is not prognostically relevant for all cancer types and all therapy types. However, to answer this question properly, further studies on larger cohorts are required.

Furthermore, we have added to or changed most manuscript figures to make the cancertype specific results more visible:
Figure 1, updated with COAD MSS & MSI subtypes
Figure 2, completely redesigned with new panels b, c, e, & f to show cancer type specific results
Figure 3, previous figure 2d, reassigned to its own figure, showing cancer type specific results
Figure 4, redesigned previous figure 3, showing cancer type specific results
Figure 5, new figure, showing cancer type specific results comparing adaptive to innate scores
Supplementary figure 4, added COAD MSS and COAD MSI
Supplementary figure 5, added COAD MSS and COAD MSI
Supplementary figure 7, added cancer type to HMF
Supplementary figure 10, added COAD MSS and COAD MSI
Supplementary figure 11, new figure comparing adaptive to innate scores in Mariathasan BLCA

While the reviewer was quite firm in their arguments, we hope the above may sufficiently alleviate their concerns so that this project may now be shared with a wider audience.

All considered, we are very appreciative of the time and effort the reviewer has placed on our manuscript, and there is no question that with the extensive revisions performed during this process the resulting manuscript and the presented analysis has been thoroughly improved. We hope that with this last round of major revisions, we may convince the reviewer that the manuscript is now in a state sufficient for publication.

Detailed comments:
- Thanks for clarifying the scaling approach. I have no concerns about the described approach.

We thank the reviewer for the comment

- This claim in the abstract is from a pan-cancer / cancer-type-blind analysis and must be replaced:
"Pan-cancer analysis of primary tumour samples from TCGA showed improved progression free survival in 30 patients with an A/I ratio above median (P < 0.0001)."

We have removed this sentence from the abstract, and replaced it with a meta-analysis that considers cancer types

"A meta-analysis of 32 cancer types from TCGA overall showed improved progression free survival in patients with an A/I ratio above median (Hazard ratio (HR) females 0.73, HR males 0.86, P < 0.05)".

- This claim in the abstract is from a pan-cancer / cancer-type-blind analysis and must be replaced: For patients with metastatic disease, we found that 33 responders to immunotherapy have a significantly higher A/I ratio than non-responders in HMF (P = 0.036).

We respectfully disagree with this comment, as here we are specifically investigating IO response within a limited group of patients. The question is relevant to ask across all samples, and the text in the main body of the manuscript goes into detail with the individual cancer types where the observation still stands. Hence, in our humble opinion, this is not misleading.

- Figure 1a still reports results ignoring cancer type. Please remove these results entirely and replace them with the results of Figure S1.

The concept behind this manuscript is that the type of immune cell infiltration matters. Figure 1A demonstrates the logical reasoning behind the project, and serves to illustrate which cell types are part of each group. The take-away message here is that some immune cell types are associated with improved outcome, others vice-versa. For full transparency, Figure S1 shows the same analysis with cancer type, here, the HRs demonstrate the same trend, though prognostically, this is unsurprisingly often not significant given the strong prognostic effect of primary cancer type, which we also clearly describe as such in the text. Overall, based on this analysis, we found that, pan-cancer, there was a tendency for the cell types to behave differently depending on which immune cell group they belonged to, compared to survival. We also found that it was different cells within the groups for different cancer types that would be significantly associated with outcome. This is illustrated very poorly by the plot including cancer types, Figure S1A, but remarkably well on the plot without them. Therefore, as this is a discovery analysis, we will argue that it is reasonable to keep the current figure, and politely disagree with the reviewers' firm opposition to this concept. However we have altered the text to ensure the reader is aware of the purpose of the analysis and the strong association of cancer type and outcome.

"When we performed the same analysis including cancer types as covariates, the same overall pattern was observed with regard to the direction of association of the individual immune cell types, although unsurprisingly cancer type was by far the most significant covariates relative to outcome reflecting established cancer-type specific prognosis (Figure S1a)."

- The COAD samples still haven't been split into MSI high and MSS. Because these subtypes are profoundly immunologically different, ignoring them makes it hard to interpret your results for this cancer type. E.g. I see males and females have very different results for COAD in Figure S1A, but I don't know if that's just confounding due to MSI status. The TCGA clinical metadata will have this field. I know that stratifying further reduces sample size, but if the alternative to an underpowered analysis is a confounded analysis, then the underpowered analysis is the right choice. This same comment applies to TNBC BRCA, but the effect these is less dramatic.

We acknowledge that the MSI status of the COAD samples is important, and we have now added the status to the TCGA patients, and rerun all the plots. What we see most clearly is that the MSS COAD patients have by far the largest gain from a high A/I ratio, and then unlabeled COAD - but still only for the male patients. For the female COAD patients none of the three groups are significant.

We have added the new results in the manuscript and added the division of the COAD patients in the methods section:

Methods:
"Cancer type abbreviations are found in Table 1. Information regarding MSI status [16] in colon cancer was used to split the COAD patients into COAD MSI, COAD MSS and COAD, the latter for the patients where the information was not available. "

Results:
"We observed that a higher A/I ratio significantly associated with improved outcome in 12 cancer types (COAD, COAD MSS, HNSC, BLCA, CESC, MESO, UCEC, BRCA, CHOL, LIHC, LUAD & LUSC), supporting the known role of the adaptive immune system in combating cancer[32]. Interestingly, for COAD, COAD MSS, HNSC, LIHC, LUAD and LUSC only males showed a significant association, while for MESO, only females."

- “We found that a high adaptive component is significantly associated with improved survival (pan-cancer: HR = 0.016, P = 9.20*10-7 173 , cancer informed: HR = 0.071, P = 0.0014,) whereas a high 174 innate component is associated with an poor prognosis, although only significantly in pan-cancer (pancancer: HR = 80.9, P= 1.94*10-6 175 , cancer informed: HR = 1.042, P= 0.57)” -> Please remove the result from the unadjusted analysis.

Here we clearly show the results for the cancer informed model, as well as the pan-cancer model, thus we politely insist that it is not unreasonable to keep the pan-cancer results.

- Thank you for the analysis including AI ratio and total TILs. I think this statement is not supported by the printed results: “indicating that the specific ratio of adaptive to innate immune cells is slightly more relevant to disease outcome than total TIL infiltration, but not for all cancer types”. Please either remove it or give a p-value for the difference between the 0.92 and 0.94 hazard ratios.

We apologise for our poor phrasing and thank the reviewer for bringing this to our attention. As both terms are significantly associated with outcome, we have changed the text as indicated below:

“When we included cancer type as a covariate in the model, both terms remained significantly associated improved outcome (AI ratio: HR = 0.92 , P = 0.000988, TIL: HR = 0.94 , P = 0.000792), indicating that both the specific ratio of adaptive to innate immune cells and the total amount of immune cells are independently associated with outcome.”

- “We observed that the AI ratio remained highly significant (AI ratio: HR = 0.77 , P < 2x10^-16). While the total TIL score was significant in univariate analysis (HR = 0.93, p = 2.95x10-7 215), it was not 216 significant in the multivariate analysis (TIL HR = 1.03, P = 0.061).” -> Please remove any reference to pan-cancer analyses ignoring tumor type.

Here we clearly show both cancer-informed and pan-cancer, thus we politely disagree with the reviewers position.

- Figure 2a: please remove, since it ignores cancer type. Moving the results of S5 to a main figure would be appropriate (or at least a summary of them, e.g. a forest plot).
- Figure 2b: please remove, since it ignores cancer type.

We have added forest plots for both TCGA and HMF, as 2b-c and 2e-f, respectively. To these we have added a meta analysis, to show that overall an A/I ratio above median leads to an improved survival. The text regarding these plots have been changed to:

“When we performed survival analysis on the combined TCGA cohort including all patients, we found that both female and male patients with an A/I ratio above median had significantly improved overall survival relative to patients with an A/I ratio below median (Figure 2a). We performed the same analysis on the individual cancer types, and found that 7/30 cancer types (BRCA, CESC, HNSC, LICH, OV, SKCM and UCEC) showed significantly improved outcome with an A/I ratio above median, while 2/30 (LGG and UVM) showed the opposite (Figure S4). Based on these results, we performed a metaanalysis which take all cancer types into account, on male and female patients separately. Here, we observed that an A/I ratio above median associated with improved outcome in both male and female patients, but with a stronger association in females (HR females 0.73, HR males 0.86, P < 0.05, Figure 2b-c).”

"Initially, we performed a survival analysis on the metastatic HMF cohort and found that both male and female patients had improved overall survival if their A/I ratio was above median (Figure 2d). We performed the same analysis on the individual cancer types, and found that 2/11 cancer types (BLCA and COAD) showed significantly improved prognosis with an A/I ratio above median, while no cancer types showed the opposite (Figure S6). When we performed a meta-analysis on male and female patients, respectively, we again found that an A/I ratio above median associated with improved outcome in both male and female patients (HR females 0.68, HR males 0.69, P < 0.05, Figure 2e-f)."

- "To investigate if the differences in survival were solely based on gender, we performed two cox proportional hazard models, one analysing survival relative to gender, and one analysing survival relative to gender and the A/I ratio. We then compared the performance of the models using a likelihood ratio test. Based on this analysis, we found that the model including the A/I ratio term significantly out-performed the simpler model including only gender (P = 4.45 * 10-9 237)" -> I'm reading this as ignoring cancer type. Please remove, or at a minimum adjust for cancer type.

This analysis was actually adjusted for cancer types, unfortunately this was not clearly written, this has been corrected now.

"To investigate if the differences in survival were solely based on gender, we performed two cox proportional hazard models, one analysing survival relative to gender, and one analysing survival relative to gender and the A/I ratio. Both models had age, stage and cancer type as covariates. "

- Figure S7: Same as above: please remove or stratify by cancer type. (I believe the Mariathasan dataset is all bladder cancer; if so, then its results can be kept.)

The Mariathasan data is all bladder cancer. We have stratified the HMF results by cancertype, and inserted these as figure 7d-f. We have already included many large supplementary figures with km plots for both the TCGA and the HMF and their many different cancer types. Here, it is logical to include S7A+B in order to also illustrate the gender difference on a pan-cancer level.

"To determine if the previously observed gender difference in cancer prognosis also affects survival within the two cohorts of CPI treated patients, we performed a survival analysis on gender. For both cohorts we found no significant difference in survival of male and and female patients treated by CPI (Mariathasan: P = 0.18, Figure S7c, HMF BLCA: P = 0.081, Figure S7d, HMF LUNG: P = 0.17, Figure S7e, HMF SKCM: P = 0.72, Figure S7f), indicating that drug-induced activation of the adaptive immune response may out-weigh any gender-specific differences in the immune response."

- Line 280: "For both cohorts we found no significant difference in 281 survival of male and and female patients treated by CPI" -> the HMF results should be stratified by cancer type.

We have stratified the HMF results by cancertype, and inserted these as figure 7d-f.

"For both cohorts we found no significant difference in survival of male and and female patients treated by CPI (Mariathasan: P = 0.18, Figure S7c, HMF BLCA: P = 0.081, Figure S7d, HMF LUNG: P = 0.17, Figure S7e, HMF SKCM: P = 0.72, Figure S7f), indicating that drug-induced activation of the adaptive immune response may out-weigh any gender-specific differences in the immune response."

- Figure 3b,d,f: please remove or stratify by cancer type.

Figure 3 is now re-labeled Figure 4. Figure 4b and 4d have been stratified by cancer type to allow for investigation of individual cancer types. Figure 4f is a summary and is kept as is.

- “This supports our findings that metastatic melanoma with a poor response to CPI have a lower A/I ratio, indicating that they have a relatively high expression of the innate immune system.” -> This is a rather indirect inference. You should either remove this statement or back it up with an analysis of the innate immune score in the relevant samples.

Thank you for this comment, we agree that we could show this in a more direct manner, and we have therefore added a new figure to show this more clearly, Figure 5, which we have inserted below for your convenience. We have added the following text to the Result section:

“To further elucidate the relationship between expression of the adaptive and innate immune system in the tumour microenvironment of the CPI treated patients with progressive disease, we investigated the adaptive vs. the innate expression scores directly. Here, we observed that for both the HMF cohort and the Mariathasan bladder cancer, a very large proportion of patients with progressive disease showed higher innate relative to adaptive scores (BLCA: 96%, LUNG: 76 %, SKCM: 83%, Figure 5. Mariathasan BLCA: 79%, Figure S11). This supports the use of a ratio as a proper tool to identify patients with relatively increased adaptive-to-innate expression, and potentially improved therapy response”

and to the Discussion:

“This supports our findings that 83% of patients with metastatic melanoma with a poor response to CPI have a lower A/I ratio, indicating that they have a relatively high expression of the innate immune system (Figure 5).”

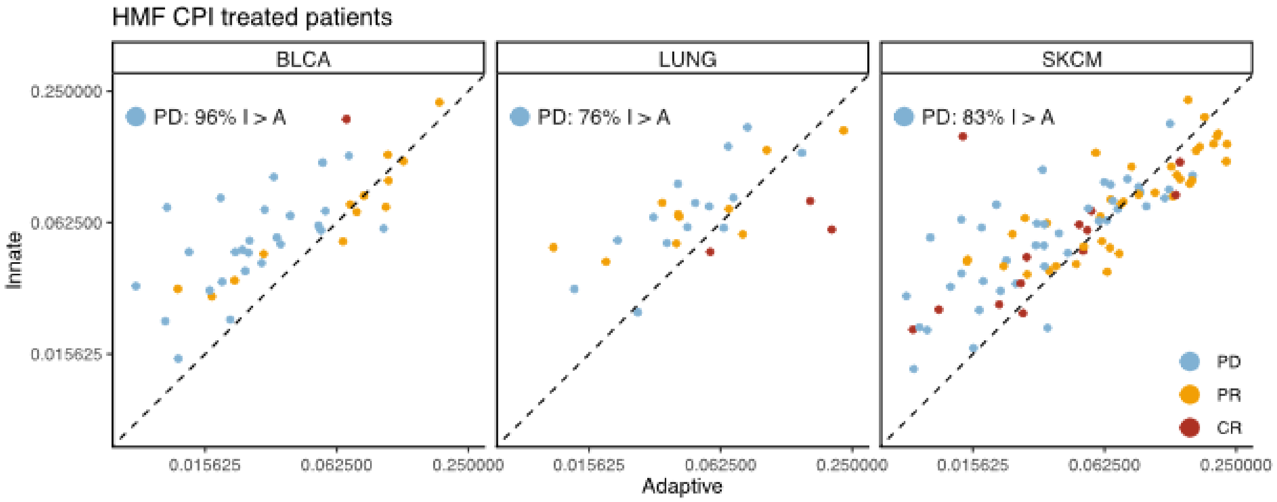

- It looks like your analyses found that innate abundance wasn’t informative when considered alongside adaptive abundance. At a minimum, please discuss the implications of this in the Discussion. Should we just be looking at adaptive abundance alone, or does your work provide additional reasons to look at A/I ratio?

It is our opinion that our work very convincingly shows that the ratio is better to utilize than the abundance alone. Considering innate and adaptive scores individually, there is no question that most information is

found in the adaptive component. However, the ratio shows a more significant association, and on the new figure 5, we demonstrate how the higher innate abundance is associated with poor response to immunotherapy. Furthermore a ratio has the benefit that it is by definition normalised within each patient, thus we get a patient specific measurement that remains robust across patients and across cancer types.
