# Supplementary material for: The ratio of adaptive to innate immune cells differs between genders and associates with improved prognosis and response to immunotherapy
Source: PLoS One. 2023 Feb 6;18(2):e0281375. doi: 10.1371/journal.pone.0281375 (PMC9901741; doi:10.1371/journal.pone.0281375)
Supplement: Attachment — Submitted filename: review.pdf [file pone.0281375.s017.pdf]

The manuscript has already been through many revisions, and thus I think it is in good shape. It reads well and the concepts, ideas and evidence are clear. The authors offer a concise introduction to immunotherapy and provide a solid base for their rationale. Furthermore, I think that enough evidence is provided in favour of the hypothesis that the type of immune cell infiltration is important for patient stratification. While more statistical power is necessary to provide final evidence, we applaud the authors' efforts to mine currently available data. Advances in machine learning and statistical extrapolation, combined with the increasing number of patient data could take this concept into a fully-fledged clinical tool in the not-too-distant future.

Before publication, we would kindly ask the author to address the following:

Could you please mention in the introduction the cell types that Adaptive immune cell types (CD8 T-cells, B-cells, CD45, Cytotoxic cells, T-cells, Th1-cells and T-regulatory cells) and innate immune cell types (Dendritic cells, Macrophages, Mast cells, Neutrophils, Natural killer cells and Natural killer CD56dim cells).

Could you please fix the following typos and grammar:

Ln 20: … "are" far from perfect

Ln 49: … "plays" an ...

Ln 68: A great amount of research is "being" performed …

Ln 82: please lowercase "immune system"

Ln 110: needs a new line to separate paragraphs

Ln 135: Please isolate in a new line the equation:

$$scaled\_celltypen = (celltypen - celltypemin) / (celltypemax - celltypemin) \quad (eq1)$$

Ln 196- why are the abbreviated cancers not in alphabetical order? Is there a significance to the current order? It would be easier to look them up in Table 1 if they were.

Line 326- I would suggest softening the claim by using the word "useful" or "efficient" instead of "proper".

Line 360- Same here. I would suggest to use the words "more accurately" instead of "properly".

Line 381 - Did you mean that " , whereas here a low frequency …"

Figure 3- some cancers are missing label. Could you create a legend that goes next to it for those missing a label?

Figure general- the margins of the figures are too small, and some of the figure text overlaps with text generated by the automatic review pdf print.
