# Supplementary material for: The ratio of adaptive to innate immune cells differs between genders and associates with improved prognosis and response to immunotherapy
Source: PLoS One. 2023 Feb 6;18(2):e0281375. doi: 10.1371/journal.pone.0281375 (PMC9901741; doi:10.1371/journal.pone.0281375)
Supplement: Attachment — Submitted filename: Rebuttal_letter.pdf [file pone.0281375.s018.pdf]

Dear reviewers,

We strongly appreciate the time spent reviewing our manuscript. We have found your comments very useful, and we hope you will find that we have addressed them adequately. In our view, your comments have helped improve our manuscript, which we are very grateful for.

Below you will find a point-by-point reply to each comment.

Reviewer 2

This manuscript presents a carefully performed pan-cancer analysis, showing that the the ratio of adaptive to innate immune cells differs between females and males and associates with improved prognosis and response to immunotherapy. Using RNAseq data, an adaptive-to-innate immune ratio (A/I ratio) was defined and it was found with high significance that primary tumour samples from TCGA showed improved progression free survival in patients with an A/I ratio above median.
This is a quite interesting observation in these data sets, as the present standards for stratifying metastatic patients for immunotherapy (for e.g. via PDL1 expression or TMB measurement) is far from perfect. Thus, the proposed A/I ratio may be an additional suitable tool to identify patients which are likely to benefit from immunotherapy using checkpoint inhibitors. The gender specific aspect is also interesting and should be more carefully addressed in future studies.

I think the manuscript is suitable for PLOS1, if the authors emphasise again that the observation is so far only based on pan-cancer data and needs further independent experimental confirmation. But as an introduction to this field of research (adaptive-to-innate immune ratio), it is certainly very exciting for others researchers as well.

Thank you very much for your comments and for reviewing our manuscript. We have added an extra paragraph to the discussion, where we emphasize the need for experimental validation.

"As the results in this study are based on publicly available pan-cancer data from heterogeneous cohorts, independent experimental validation would be necessary to further validate using a ratio of adaptive to innate immune cells for patient stratification."

Reviewer 3

The manuscript has already been through many revisions, and thus I think it is in good shape. It reads well and the concepts, ideas and evidence are clear. The authors offer a concise introduction to immunotherapy and provide a solid base for their rationale. Furthermore, I think that enough evidence is provided in favour of the hypothesis that the type of immune cell infiltration is important for patient stratification. While more statistical power is necessary to provide final evidence, we applaud the authors' efforts to mine currently available data. Advances in machine learning and statistical extrapolation, combined with the increasing number of patient data could take this concept into a fully-fledged clinical tool in the not-too- distant future.

Before publication, we would kindly ask the author to address the following:

Could you please mention in the introduction the cell types that Adaptive immune cell types (CD8 T-cells, B-cells, CD45, Cytotoxic cells, T-cells, Th1-cells and T-regulatory cells) and innate immune cell types (Dendritic cells, Macrophages, Mast cells, Neutrophils, Natural killer cells and Natural killer CD56dim cells).

Thank you for this very sensible suggestion, we humbly apologise for not already having included such a section. We agree that it improves understanding to have these mentioned in the introduction. We have therefore added the following paragraphs to the introduction, and included two new references (11 and 12).

"The immune system can roughly be divided into two major branches, the innate and the adaptive. The innate immune system is our first line of defence, but it is non-specific and its primary role is to initiate inflammation when recognizing foreign pathogens, and to use phagocytosis to engulf foreign molecules and cells, and then present antigens from these to the cells of the adaptive immune system that can activate a specific immune response[11]. The adaptive immune system contains cells that undergo recombination to create unique receptors which bind to foreign peptides or peptides not usually presented by normal, healthy cells[12]."

"For this study, Dendritic cells, Macrophages, Mast cells, Neutrophils, Natural killer cells and Natural killer CD56dim cells were all analysed as part of the innate immune system. Likewise CD8 T-cells, B-cells, CD45, Cytotoxic cells, T-cells, Th1-cells and T-regulatory cells were all analysed as part of the adaptive immune system [11], [12]."

Could you please fix the following typos and grammar:

Ln 20: ... "are" far from perfect

This has been corrected.

Ln 49: ... "plays" an .. .

This has been corrected.

Ln 68: A great amount of research is "being" performed ...

This has been corrected.

Ln 82: please lowercase "immune system"

This has been corrected.

Ln 110: needs a new line to separate paragraphs

New lines have been added both above and below, to separate the three separate paragraphs.

Ln 135: Please isolate in a new line the equation:

scaled_celltypen = (celltypen - celltypemin) / (celltypemax - celltypemin) (eq1)

This has been correctly formatted as an equation.

Ln 196- why are the abbreviated cancers not in alphabetical order? Is there a significance to the current order? It would be easier to look them up in Table 1 if they were.

We have changed the order to be alphabetical. They were ordered from lowest to highest p-value, the same order in which they appeared on the figure, but we agree that it is easier to look them up, when they are in alphabetical order.

Line 326- I would suggest softening the claim by using the word “useful” or “efficient” instead of “proper”.

Thank you for this suggestion, we changed the word to “efficient”, as we agree that this better expresses the meaning of the sentence.

Line 360- Same here. I would suggest to use the words “more accurately” instead of “properly”.

Thank you for this suggestion, we have changed the words to “more accurately”, as we again agree that this better expresses the meaning of the sentence.

Line 381 - Did you mean that “ , whereas here a low frequency ...”

No, that sentence was referring to the findings in the paper, and to make this clear we have changed the sentence to

“A study reports that the baseline circulating myeloid-derived suppressor cells (MDSC) correlate with outcome of ipilimumab treatment[45], they find that a low frequency correlates to improved outcome.”

Figure 3- some cancers are missing label. Could you create a legend that goes next to it for those missing a label?

We have only labelled the significantly different cancer types. A legend has been added, to show the colour of all cancers, as we agree that this improves the understanding of the figure.

Figure general- the margins of the figures are too small, and some of the figure text overlaps with text generated by the automatic review pdf print.

Thank you for noticing this! We have made all the margins wider, to avoid any overlaps.
