# Supplementary material for: The ratio of adaptive to innate immune cells differs between genders and associates with improved prognosis and response to immunotherapy
Source: PLoS One. 2023 Feb 6;18(2):e0281375. doi: 10.1371/journal.pone.0281375 (PMC9901741; doi:10.1371/journal.pone.0281375)
Supplement: Attachment — Submitted filename: Response_to_Reviewers.docx [file pone.0281375.s014.docx]

Dear reviewer,

First of all, we would like to thank you for taking the time to revise our paper. We greatly appreciate this. We have found your comments useful, and followed them to make the paper even better. Below is a point by point list of our responses to each of them.

Best Regards,

Nicolai J. Birkbak & Johanne Ahrenfeldt

Reviewer #1: The authors investigate an important question in immune-oncology: does the balance between innate and adaptive immunity hold important prognostic/predictive information? They investigate this questions in 3 publicly-available datasets, including TCGA. The approach of looking at adaptive /immune ratio is interesting and worthwhile, and the analyses are rich. However, there are flaws in the statistical analyses that make many of the paper’s key results unsupported. If the analyses are corrected, presumably causing many key results to change, then this manuscript could be a worthy addition to the literature.

Major concern 1:

Many of the study’s key statistical models do not consider cancer type, leaving them subject to confounding and erroneous results. These models must be corrected for the paper’s conclusions to be believed. The minor concerns below expand on this topic.

We apologise for this oversight. We have now performed all models on each cancer type individually or with the cancer types as covariates (particularly note the updated supplementary figure 1). We are happy to report that this has not in any major way changed our results, in most cases the A/I ratio remains significantly associated with outcome. In some cancer types, the association is not significant or less significant, but here the trend mostly remains, and some of this is likely a power issue.

Major concern 2:

The standard model of immune-oncology would hold that, “adaptive immune cells are helpful for survival.” With its focus on adaptive/innate ratio, this paper claims that innate cells hold additional information. But this claim is never directly evaluated. (There is a multivariate analysis of A/I ratio and total TILs, but I believe total TILs includes both innate and adaptive populations.) To see whether innate cells have any prognostic information to add, multivariate models should be run with both halves of the A/I score. Then we could see whether adaptive cells are the whole story, or whether innate cells have a countervailing effect. Unless this analysis is done, I’m left to wonder whether A/I ratio is really only meaningful insofar as it provides an oblique readout of total adaptive immunity.

We appreciate this insightful comment from the reviewer. This is indeed a very good point. Based on this suggestion, we have made a multivariate model where we test for both adaptive and innate immune cells as covariates This has been done with and without cancer types as covariates in the model. Omitting cancer types, we see a very striking result, where both innate and adaptive immune cells are significantly associated with outcome in opposite directions (Adaptive HR =  0.016, P =  9.20*10^-7, Innate HR = 80.9, P=  1.94*10^-6). Including cancer types in the model, we observe the same signal, albeit weaker  (Adaptive HR =  0.071, P =  0.0014, Innate HR = 1.042, P=  0.57).

We have included this analysis in the manuscript:

*“To evaluate if the two compartments of the immune system do indeed pull in opposite directions when it comes to patient outcome, we used a multivariate model, where the innate and the adaptive values were covariates together with age, stage and gender. This was done both pan-cancer and including the cancer types as covariates. We find that a high adaptive component is significantly associated better survival (pan-cancer: HR = 0.016, P =  9.20*10^-7^, cancer informed: HR =  0.071, P =  0.0014,) where as a high innate component is associated with an poor prognosis, although only significantly in pan-cancer (pan-cancer: HR = 80.9, P=  1.94*10^-6^, cancer informed: HR = 1.042, P=  0.57).*”

More minor concerns are below:

The approach of contrasting innate and adaptive scores is interesting and sound. (Not a concern.)

Line 72: “macrophages are the most abundant” – add a reference please.

Our apologies, the reference for this was inserted further below. We have now moved the reference up to the end of this sentence. Furthermore the sentence was changed to “Tumour associated macrophages are one of the most abundant cells in the TME [(Singh et al. 2019)](https://paperpile.com/c/DK23M7/j9HA)”

The text needs some light editing for spelling, grammar, and conciseness.

Line 106: only PD, PR and CR were used. Were Stable Disease cases omitted then? If so, why? Without a justification, this looks like cherry-picking the data.

Thank you for pointing this out. Not including our justification was an oversight. We have now added our reasons behind this decision. Stable disease is specifically not included in the analysis, as it is not clearly associated with either response or disease progression. Patients with stable disease may have extended survival can be considered as experiencing clinical benefit from treatment, even if the tumour does not shrink following therapy. To make this clear in the manuscript, we have added the following to the methods:

“*Patients with stable disease (SD) were not included in the analysis, as the interpretation of SD is not clearly defined as good or poor outcome. Indeed, it can be both a sign that the therapy works and contains tumour growth, or it can be a sign that the therapy has no effect but the tumour size remains unchanged due to stagnated growth*”

117: “a linear scaling was performed” – please give the full details of this operation, and the motivation. Because the cell type scores are log-scale, some scaling approaches might lead to strange interpretations, while simply mean-centering would retain the log-scale interpretation of the scores.

We wanted to have all of the values between 0 and 1, for easy interpretation of the calculation. This makes the value for all cell types comparable, and we can then sum them up and use their mean. All values were un-logged prior to scaling. For each cell type the maximum and minimum value was found, and the scaled value was calculated as scaled = (unscaled - minimum ) / (maximum - minimum).

We have changed the text in the methods to describe this in more detail:

*“A linear scaling of the expression values for each cell type was performed as follows, first the values were reverse log-transformed, and then the values within each cell type were linearly scaled to values between 0 and 1, with this equation: scaled_celltype_n_ = (celltype_n_ - celltype_min_ ) / (celltype_max_ - celltype_min_), and then a mean scaled expression a score for each group (adaptive and innate) was calculated per sample as the mean scaled value for the cell types within the group per sample, whereafter the A/I ratio was determined by dividing the adaptive with the innate score.”*

Figure 1a: Fitting a single model to all cancer types doesn’t make much sense. In particular, I’m worried that cancer type acts as a powerful confounder in the analysis – e.g. melanoma is both highly infiltrated and deadly, confounding the relationship between immune abundance and survival. Secondly, it seems hard to justify the assumption that immune cells have the same survival implications in each cancer type. (For example, in glioblastoma, I believe high immune abundance predicts poor survival). To address these concerns, please run this analysis separately for each cancer type and report those results instead. And ideally, BRCA would be split into HR+ vs. TNBC, and COAD would be split into MSI-high vs. MSS.

When we modelled on single cancer types, or added cancer types as a covariate, most cell types were not individually significant, occasionally except for Macrophages, Mast cells and NK cells. Thus, to leverage the power of multiple cell types with correlated abundance, we decided to investigate the ratio of adaptive to innate immune cells. To make this more clear, we have now added a forest plot from the model with cancer types as covariates to Supplementary figure 1a, and expanded on our reasoning behind the AI-ratio in the explanatory text:

“*We then fitted a multivariable Cox proportional hazard model to the progression free interval, including all immune cell types and gender, age and tumour stage as covariates (Figure 1A omitting age, stage and gender from the visualisation. Full results with all covariates listed in Table S2). Of 14 immune cell types, 8 showed a significant association with outcome, four with improved survival, four with worse survival. Overall, we observed that adaptive immune cells associated with a lower risk of relapse (CD8 T-cells, CD45, T-cells, Th1 cells, Treg), while innate immune cells was associated with a higher risk of relapse (Dendritic cells, Macrophages, Natural killer cells) (Figure 1a). When we performed the same analysis including cancer types as covariates, the same overall pattern was observed (Figure S1a). To further evaluate how the two compartments of the immune system associate with patient outcome in opposite directions, we divided the cell types based on which of the two major immune components they belong to and calculated a value for each component. We then performed a multivariate model including the innate and the adaptive values together with age, stage and gender. This was done separately pan-cancer and with cancer type as covariates. We found that a high adaptive component is significantly associated with improved survival (pan-cancer: HR = 0.016, P =  9.20*10^-7^, cancer informed: HR = 0.071, P =  0.0014,) whereas a high innate component is associated with an poor prognosis, although only significantly in pan-cancer (pan-cancer: HR = 80.9, P=  1.94*10^-6^, cancer informed: HR = 1.042, P=  0.57).*”

Additionally, while we agree that splitting BRCA and COAD into subtypes would be optimal, this also reduces the power of the cohorts.

Figure 1a: in addition, please expand your regressions to adjust for other variables relevant to survival, e.g. tumor grade, stage, sex, age, etc…

The model used to produce figure 1a, do already contain age, sex and stage. To improve clarity, we have added more detail to the text where we explain this:

“*We then fitted a multivariable Cox proportional hazard model to the progression free interval, including all immune cell types and gender, age and tumour stage as covariates (Figure 1A omitting age, stage and gender from the visualisation. Full results with all covariates listed in supplementary table 2)*

The TCGA datasets DLBC and LAML (and possibly THYM) are tricky for immune decomposition, as these immune-driven cancers express many immune marker genes. I recommend removing them to ensure they don’t contaminate the more reliable results from solid tumors.

We thank the reviewer for this insightful comment. We agree that is a valid point, and have removed these three cancer types from the analysis.

Please define how “total TIL infiltration” is calculated.

We apologise for the lack of clarity. We have expanded the text describing how the total TIL infiltration is calculated in the methods section, pasted here below:

“*Tumour immune cell decomposition was performed using the score defined by Danaher and colleagues*[*^21^*](https://paperpile.com/c/DK23M7/AErVe) *based on whole tumour RNAseq data, implemented as described*[*^22^*](https://paperpile.com/c/DK23M7/Yikcb)*. We used a defined list of genes from Danaher*[*(Danaher et al. 2017)*](https://paperpile.com/c/lVVn4G/w9ui) *to define the expression of immune cell types, and the mean of the cell types described in the paper was then used as the total TIL score*”

Figure 1d: a forest plot might show these results to better effect. For example, is the COAD hazard ratio for males significantly different than it is for females? A forest plot would show this, while the volcano plot cannot.

We thank the reviewer for this suggestion. While we specifically chose a volcano plot as we believe it is a more illustrative method to show the effect of gender, we have now also made a forest plot version. Both methods of course show the same results, but we are respectfully of the opinion that the volcano plot is more illustrative. Nevertheless, we have included the forest plot as Supplementary Figure 1b.

Figure 1e: can you label the low outlier cancer type? That seems interesting.

We agree this is of interest and thank the reviewer for the suggestion. We have added labels to the outliers.

“…the specific ratio of adaptive 161 to innate immune cells is more relevant to disease outcome than total TIL infiltration.” This claim is both important and novel, and so it deserves to be backed up more thoroughly.

Very important: the regression of survival on A/I and TILs should be run separately for each cancer type; otherwise cancer type is a confounder.

We thank the reviewer for the nice comments and the suggestion, we have performed the analysis, and we have changed the paragraph in the paper so reflect the added analyses:

*“While the total TIL score was significant in univariate analysis (HR = 0.93, p = 2.95x10^-07), it was not significant in the multivariate analysis (TIL HR = 1.032, P = 0.06114). However when we run this model with cancetype as a covariate, both terms are associated with significantly better outcome (AI ratio: HR = 0.92 , P = 0.000917, TIL: HR = 0.94 , P = 0.002245), indicating that the specific ratio of adaptive to innate immune cells is slightly more relevant to disease outcome than total TIL infiltration, but not for all cancer types.”*

Figure 2a: same concern: a model combining all cancer types together is probably invalid. Please re-run this model separately for each cancer type.

Thank you for this comment, we agree that the cancer types are important to incorporate in the analysis.  We have now performed the analysis, the results are in a supplementary figure 4.

Lines 167-171: This argument ignores how different cancer types have different incidence in males and females. E.g. breast cancer, the biggest TCGA dataset, occurs mainly in females. Thus the argument does not support the conclusion that “This suggests that some of the 171 gender difference in survival can be predominantly explained by the increased A/I ratio in females.” Instead, you could model survival vs. sex, then again vs. sex + A/I ratio. The change in sex’s effect size between the models would get at how much of sex effects are explained by A/I. (As with all the other models, this analysis would have to be performed per cancer type.)

This model has been performed for each cancer type individually, and cancer types where there are mainly female patients have been excluded from this analysis, so Breast, Cervix  and Ovarian cancer are not part of this analysis. Neither is Prostate cancer, as there are no females to compare with.

We have followed the suggestion to model survival vs. gender and compared it to survival vs. gender + A/I ratio. And we did this with the cancer types as covariates. The results show that the model with our A/I ratio is a significantly better model than only gender. This has been added to the results:

*“To investigate if the differences in survival were solely based on gender, we performed two cox proportional hazard models, one analysing survival relative to gender, and one analysing survival relative to gender and the A/I ratio. We then compared the performance of the models using a likelihood ratio test. Based on this analysis, we found that the model including the A/I ratio term  significantly out-performed the simpler model including only gender (P = 4.45 * 10^-9^).*”

Line 178, “This suggests that a high A/I ratio may lead to a lower frequency of patients progressing to 179 metastatic disease.” That’s making too strong a conclusion from the available data – there are all sorts of reasons different study populations could differ. Please remove this claim or support it more rigorously.

We apologise the strong language, we have rephrased this section as:

*“Taken together, this suggests that a high A/I ratio may be one of the factors that contribute to a lower frequency of patients progressing to metastatic disease.”*

Figure 2b: please clarify which cohort this analysis is from.

We apologise for the oversight, we have improved the text describing Figure 2 as:

*“Next, we performed a survival analysis on the metastatic HMF cohort and found that both male and female patients had improved overall survival if their A/I ratio was above median (figure 2b)”*

Additionally, the cohort is mentioned on the figure itself (“HMF”), and in the figure legend

Figure 2b: please stratify by cancer type

Apologies, we have made the figure for each individual cancertype, these have been added as supplementary figure 6.

Figure S5b: please stratify by cancer type

We have stratified based on cancertype, and added this as supplementary figure 10.

Line 279-281: “This suggests that immunotherapy may be relatively more effective in males than females, 280 with males achieving greater benefit from immunotherapy compared to chemotherapy or targeted 281 therapies.” This is phrased too strongly, unless you can back it up with some statistics (including confidence intervals).

Thank you for pointing this out, we have tried and failed to phrase it less strongly or to find a way to show this statistically, but instead we have ended up removing this sentence, as we do not have sufficient data to back it up.

Additional Editor Comments:

Although it remains unclear whether the analysis of the RNAseq data might really allow determining the host immune response within a cancer, the sole fact that a pattern of protein expression associated with immune functions demonstrated correlation with the outcome of immunotherapy is of significant importance.

The authors should enter the abbreviation list for the different cancer types within the manuscript and not only in the supplement.

We have added a list of abbreviations as Table 1.

It could be helpful to include a table with the main genes which allow differentiation between innate and adaptive immunity.

Thank you for this input, we have added the genes as Supplementary Table 1.

In addition, the authors should provide information for all analyzed cancer types on gene expression of proteins involved in immune functions that might be expressed by the cancer itself (for example based on cell culture experiments) and not by infiltrating immune cells (such as IL6 in malignant melanoma), as this might constitute a confounding factor.

The possible expression of these proteins by cancer cells should be discussed within the discussion section.

Thank you for this very good point, we agree that this does add a layer of nuance to the discussion. We therefore used data from the CCLE (Cancer Cell Line Expression) project downloaded from DepMap (DepMap, Broad (2022): DepMap 22Q2 Public. figshare. Dataset. <https://doi.org/10.6084/m9.figshare.19700056.v2>) to evaluate the expression of immune genes. For each sample the genes were ranked based on expression values (low rank = low expression), and for each cancer type, we calculated the mean rank score per gene. We did the same for TCGA, and in supplementary figure 3 we now show the values for the cancer types where data from both datasets were available.

We have added the following to the results:

“*To confirm that the expression of immune related genes are in fact originating from the TME and not from the cancer cells we explored the expression of the individual immune genes in the Cancer Cell Line Encyclopedia (CCLE)^29^, a dataset of cancer cell lines (thus devoid of any infiltrating immune cells). Here we observed that the ranked expression of the immune genes were low for all cancer cell lines, except for cell lines originating from leukaemia and lymphoma, both cancers of the immune system. When we compared the ranked expression of cancer cell lines to TCGA tumour samples of matched tissue, we observed significantly higher ranks for all tumours (Figure S3), indicating that the observed immune signal is indeed originating from infiltrating immune cells, and is not from cancer cells expressing immune-related genes.*”

And the following to the discussion:

“*To ensure that the immune gene expression signal we see from the A/I ratio does indeed come from the immune cells in the TME and not from the cancer itself, we compared the ranked gene expression from the tumour samples from TCGA, which contain both cancer cells and cells from the surrounding tissue, to the cancer cell line samples from the CCLE project^29^, that only contain cancer cells. We found that the expression of immune genes are consistently much lower in the cancer cells, median in the bottom 25 %, than in the mixed cells, median in the top 50 %. Based on these results, we are confident that the immune signal we observe in our data analysis originates from the infiltrating immune cells found within the TME.*”
