# Supplementary material for: The ratio of adaptive to innate immune cells differs between genders and associates with improved prognosis and response to immunotherapy
Source: PLoS One. 2023 Feb 6;18(2):e0281375. doi: 10.1371/journal.pone.0281375 (PMC9901741; doi:10.1371/journal.pone.0281375)
Supplement: Attachment — Submitted filename: Reviewer Comment for PONE-D-22-08886.docx [file pone.0281375.s016.docx]

This manuscript presents a carfully performed pan-cancer analysis, showing that the the ratio of adaptive to innate immune cells differs between females and males and associates with improved prognosis and response to immunotherapy. Using RNAseq data, an adaptive-to-innate immune ratio (A/I ratio) was defined and it was found with high significance that primary tumour samples from TCGA showed improved progression free survival in patients with an A/I ratio above median.

This is a quite interesting observation in these data sets, as the present standards for stratifying metastatic patients for immunotherapy (for e.g. via PDL1 expression or TMB measurement) is far from perfect. Thus, the proposede A/I ratio may be an additional suitable tool to identify patients which are likely to benefit from immunotherapy using checkpoint inhibitors. The gender specific aspect is also interesting and should be more carefully adressed in future studies.

I think the manuscript is suitable for PLOS1, if the authors emphasise again that the observation is so far only based on pan-cancer data and needs further independent experimental confirmation. But as an introduction to this field of research (adaptive-to-innate immune ratio), it is certainly very exciting for others researchers as well.
